# Supplementary material for: TNFα drives pulmonary arterial hypertension by suppressing the BMP type-II receptor and altering NOTCH signalling
Source: Nat Commun. 2017 Jan 13;8:14079. doi: 10.1038/ncomms14079 (PMC5241886; doi:10.1038/ncomms14079)
Supplement: Supplementary Information — Supplementary Figures and Supplementary Tables. [file ncomms14079-s1.pdf]

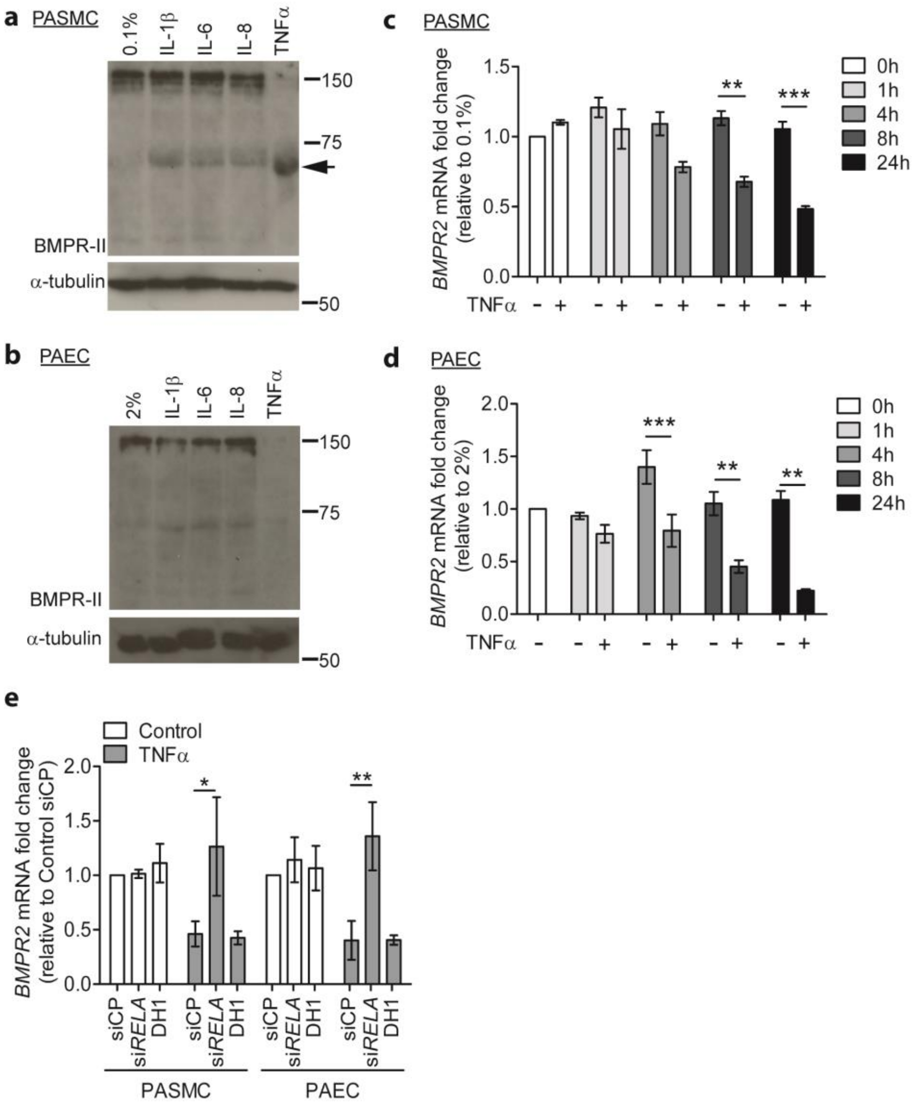


**Supplementary Figure 1. TNFα reduces BMPR-II protein and mRNA expression via NF-κB/*RELA*.** (**a** and **b**) Representative immunoblots of BMPR-II in human dPASMCs (**a**) and PAECs (**b**) treated with IL-1β (1 ng $mL^{-1}$), IL-6 (25 ng $mL^{-1}$), IL-8 (25 ng $mL^{-1}$) and TNFα (1 ng $mL^{-1}$) for 24 h. Blots were reprobed for α-tubulin to ensure equal loading. The data shown are representative of three experiments. (**c** and **d**) *BMPR2* mRNA expression, normalized to *ACTB*, in (**c**) human dPASMCs and (**d**) PAECs treated with TNFα (1 ng $mL^{-1}$) for 1, 4, 8 or 24 h ($n$ = 3; Student's $t$-test). (**e**) *BMPR2* mRNA expression, normalized to *ACTB*, in human control dPASMCs and PAECs transfected with DharmaFECT1™ alone (DH1), si*RELA* or non-targeting siRNA control (siCP). Cells were treated with TNFα (1 ng $mL^{-1}$) for 24 h ($n$ = 3; Student's $t$-test). $*P \leq 0.05$, $**P \leq 0.01$, $***P \leq 0.001$. Error bars represent mean ± s.e.m. Lower molecular mass BMPR-II is indicated by arrow.

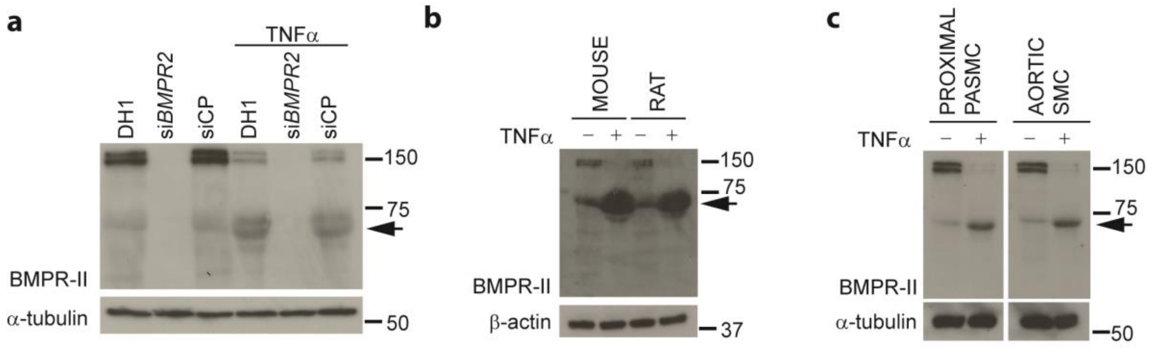


**Supplementary Figure 2. TNFα reduces BMPR-II protein and generates a cleavage product in SMCs.** (**a**) Representative immunoblots of BMPR-II in human control dPASMCs transfected with DharmaFECT1$^{TM}$ alone (DH1), si*BMPR2* or non-targeting siRNA control (siCP). Cells were treated with TNFα (1 ng mL$^{-1}$) for 24 h. Blots were reprobed for α-tubulin to ensure equal loading. The data shown are representative of three experiments. (**b**) Representative immunoblots of BMPR-II in mouse and rat PASMCs treated with TNFα (1 ng mL$^{-1}$) for 24 h. Reprobed for β-actin to ensure equal loading. The data shown are representative of three experiments. (**c**) Representative immunoblots of BMPR-II in human control proximal and aortic PASMCs treated with TNFα (1 ng mL$^{-1}$) for 24 h. Blots were reprobed for α-tubulin to ensure equal loading. The data shown are representative of three experiments. Lower molecular mass BMPR-II is indicated by an arrow.

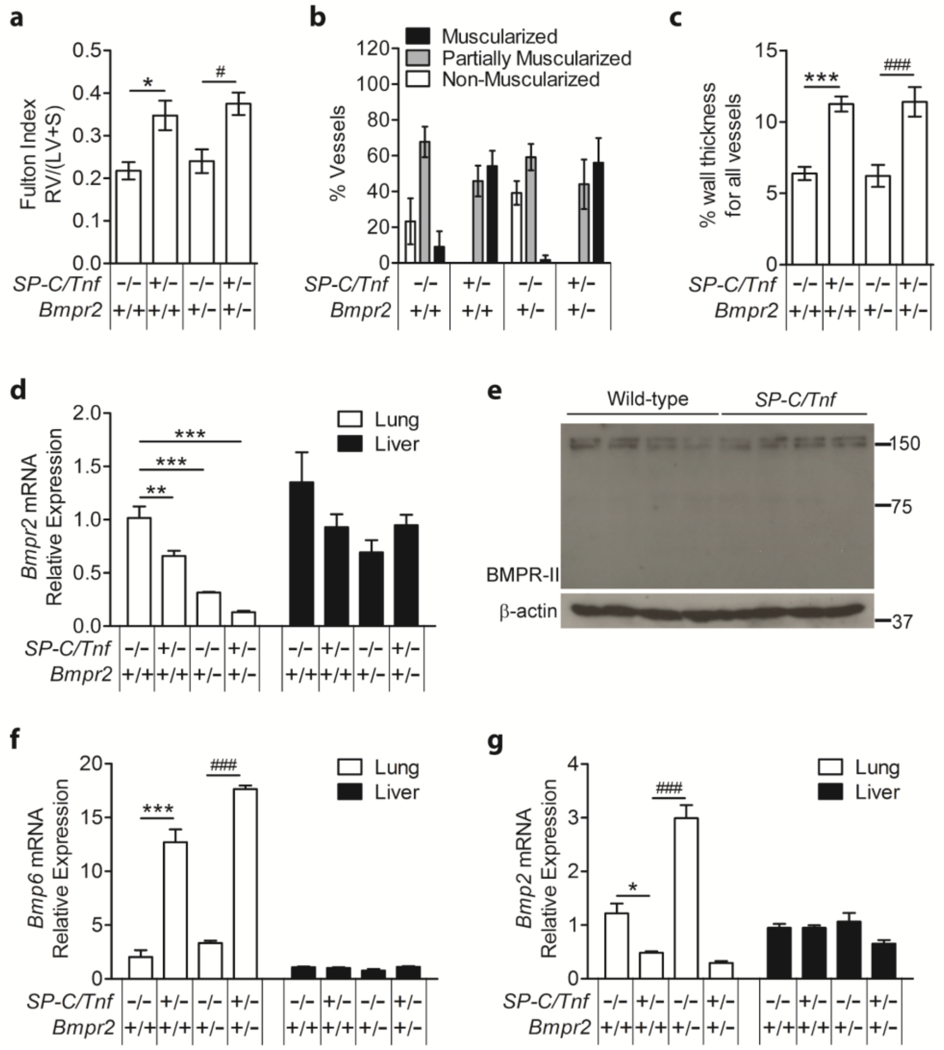


**Supplementary Figure 3. Lung specific expression of TNFα induces pulmonary hypertension.** *Bmpr2*$^{+/+}$, *SP-C/Tnf x Bmpr2*$^{+/+}$, *Bmpr2*$^{+/-}$ and *SP-C/Tnf x Bmpr2*$^{+/+}$ ($n$ = 4 of each) transgenic mice were assessed at 8-9 weeks old. (**a**) Assessment of right ventricular hypertrophy (Fulton index (RV/LV+S)). (**b**) Quantification of the numbers of non-, partially and fully muscularized arteries as a percentage of total alveolar wall and duct arteries ($n$ = 6 for all groups). (**c**) Assessment of pulmonary arterial wall thickness as a percentage of luminal diameter ($n$ = 6 for all groups). (**d**) *Bmpr2* mRNA expression, normalized to *Actb*, in lungs and livers isolated from the above mice. (**e**) Representative immunoblot of BMPR-II expression in livers isolated from 8-9 week old *Bmpr2*$^{+/+}$ and *SP-C/Tnf x Bmpr2*$^{+/+}$ transgenic mice. Blots were reprobed for β-actin to ensure equal loading ($n$ = 4). (**f**) *Bmp6* and (**g**) *Bmp2* mRNA expression, normalized to *Actb*, in lungs and livers isolated from the above mice. One-way ANOVA with post-hoc Tukey's for multiple comparisons used in **a**, **c**, **d**, **f** and **g**. */#$P \leq 0.05$, **$P \leq 0.01$, ***/###$P \leq 0.001$. Error bars represent mean ± s.e.m.

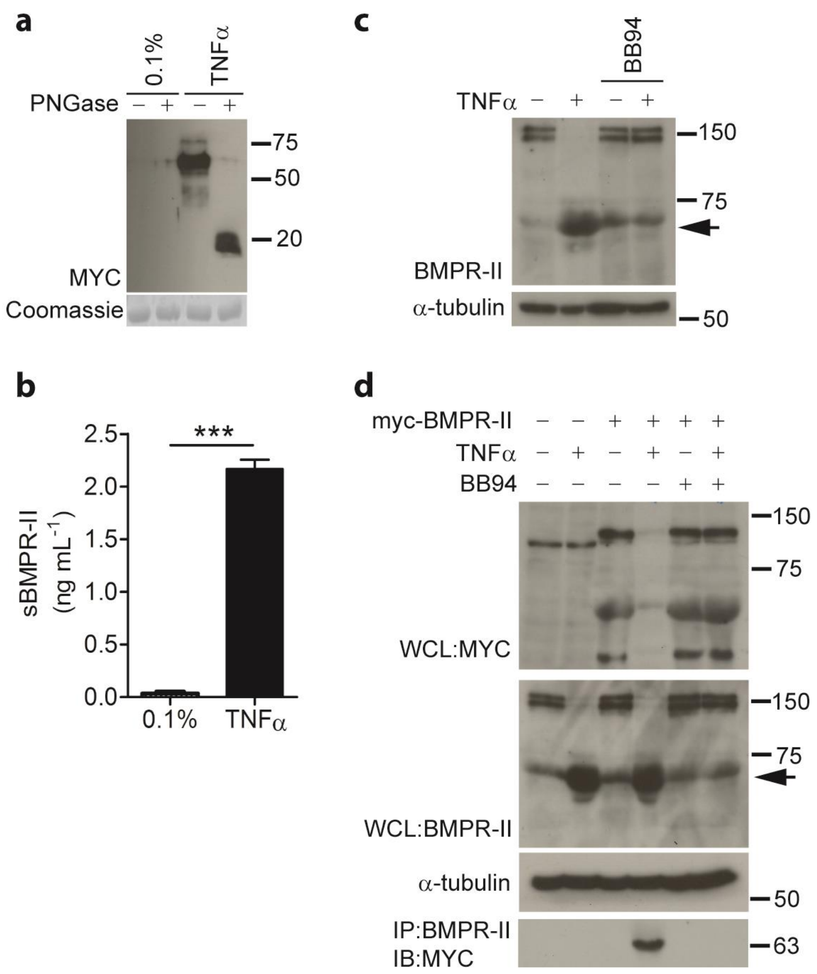


**Supplementary Figure 4. Release of sBMPR-II can be inhibited by a pan-metalloprotease inhibitor.** (**a**) Representative immunoblot of immunoprecipitated sBMPR-II in conditioned media from control human dPASMCs transfected with 5'-myc-tagged BMPR-II and treated with TNFα (1 ng mL$^{-1}$) for 24 h. Samples were deglycosylated with PNGaseF as indicated. Myc expression was assessed using immunoblotting. To ensure equal loading 0.1% FBS was loaded and assessed by Coomassie blue. (**b**) Assessment of sBMPR-II release by ELISA using control human dPASMCs treated with TNFα (1 ng mL$^{-1}$) for 24 h ($n$ = 3; Student's $t$-test). (**c**) Representative immunoblot of BMPR-II expression of control human dPASMCs pre-treated with batimastat (BB94) (10 ng mL) for 30 m prior to TNFα (1 ng mL$^{-1}$) treatment for 24 h. (**d**) Representative immunoblots of BMPR-II, myc and immunoprecipitated sBMPR-II in conditioned media from control human dPASMCs transfected with 5'-myc-tagged BMPR-II and pre-treated with batimastat (BB94) (10 ng mL$^{-1}$) for 30 min prior to 24 h TNFα (1 ng mL$^{-1}$) treatment. Blots were reprobed for α-tubulin to ensure equal loading. The data shown are representative of three experiments. ***$P \leq 0.001$. Error bars represent mean ± s.e.m. Lower molecular mass BMPR-II is indicated by an arrow.

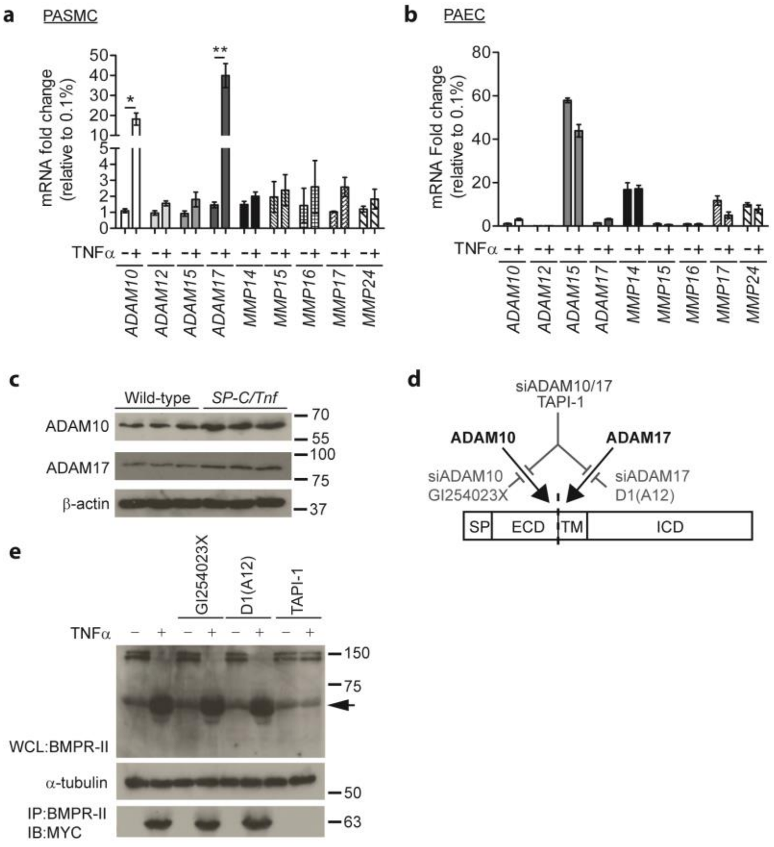


**Supplementary Figure 5. TNFα induces *ADAM10/ADAM17* expression, promoting BMPR-II cleavage in SMCs.** (**a** and **b**) *ADAM10*, *ADAM12*, *ADAM15*, *ADAM17*, *MMP14*, *MMP15*, *MMP16*, *MMP17* and *MMP24* mRNA expression, normalized to *ACTB*, in human control dPASMCs (**a**) and PAECs (**b**) treated with TNFα (1 ng $mL^{-1}$) for 24 h. ($n$ = 4; Student's *t*-test). (**c**) Representative immunoblots of ADAM10 and ADAM17 expression in lungs isolated from 8 week old $Bmpr2^{+/+}$ ($n$ = 3) and *SP-C/Tnf/*$Bmpr2^{+/+}$ ($n$ = 3) transgenic mice. Blots were reprobed for β-actin to ensure equal loading. (**d**) Schematic of pharmacological and siRNA inhibition of ADAM10 and 17. (**e**) Representative immunoblots of BMPR-II, myc and immunoprecipitated sBMPR-II in conditioned media from control human dPASMCs transfected with 5'-myc-tagged BMPR-II and pre-treated with GI254023X (10 μM), D1(A12) (50 nM) or TAPI-1 (10 μM) for 30 min prior to 24 h TNFα (1 ng $mL^{-1}$) treatment. Blots were reprobed for α-tubulin to ensure equal loading. Data are representative of three experiments. *$P \leq 0.05$, **$P \leq 0.01$. Error bars represent mean ± s.e.m. Lower molecular mass BMPR-II is indicated by an arrow.

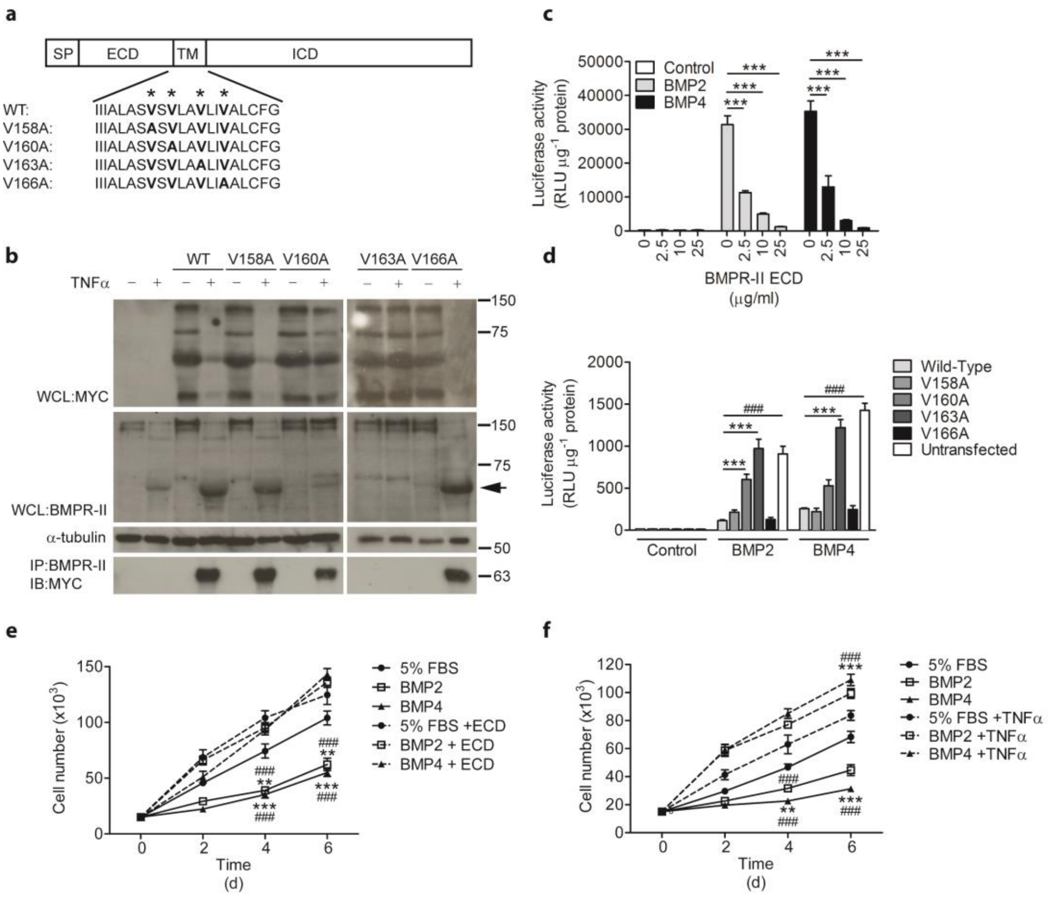


**Supplementary Figure 6. Soluble BMPR-II acts as a ligand trap.** (**a**) Schematic of full length BMPR-II (SP – signal peptide; ECD – ectodomain; TM – transmembrane; ICD - intracellular cytoplasmic domain). Valines (in bold) in 5’-myc-BMPR-II were converted to alanine (in bold) generating the V158A, V160A, V163A and V166A constructs (positions marked with “*”). (**b**) Representative immunoblots of BMPR-II, myc and immunoprecipitated sBMPR-II in conditioned media from control human dPASMCs transfected with wild-type and mutant 5’-myc-tagged BMPR-II constructs and treated with TNFα (1 ng $mL^{-1}$) for 24 h. Blots were reprobed for α-tubulin to ensure equal loading. The data shown are representative of three experiments. (**c** and **d**) Ligand trap assessment by measuring luciferase activity in C2C12-BRE cells. (**c**) C2C12-BRE cells were treated with a commercial glycosylated BMPR-II ectodomain (BMPR-II ECD) (2.5, 10 and 25 μg $mL^{-1}$) and BMP2 or BMP4 (10 ng $mL^{-1}$) for 1 h prior to measuring luciferase activity ($n$ = 3). (**d**) C2C12-BRE cells were treated with conditioned media from control human dPASMCs transfected with wild type and the mutant 5’-myc-tagged BMPR-II constructs and treated with TNFα (1 ng $mL^{-1}$) for 24 h. C2C12-BRE cells were treated with conditioned media and BMP2 or BMP4 (10 ng $mL^{-1}$) for 1 h prior to measuring luciferase activity. ($n$ = 3). (**e** and **f**) Proliferation assessment of human proximal PASMCs. (**e**) Cells were treated BMP2 or BMP4 (10 ng $mL^{-1}$) +/- BMPR-II-ECD (25 μg $mL^{-1}$) every 48 h for 6 days. Cells were counted on days 0, 2, 4 and 6 ($n$ = 3; Student’s $t$-test, **$P \leq 0.01$, ***$P \leq 0.001$ compared with 5% FBS; ###$P \leq 0.001$ compared with 5% FBS + BMPR-II ECD). (**f**) Cells were treated BMP2 or BMP4 (10 ng $mL^{-1}$) +/- TNFα (1 ng $mL^{-1}$) every 48 h for 6 days. Cells were counted on days 0, 2, 4 and 6 ($n$ = 3; Student’s $t$-test, **$P \leq 0.01$, ***$P \leq 0.001$ compared with 5% FBS; ###$P \leq 0.001$ compared with 5% FBS + TNFα). One-way ANOVA with post-hoc Tukey’s for multiple comparisons used in **c** and **d**. ***/###$P \leq 0.001$. Error bars represent mean ± s.e.m. Lower molecular mass BMPR-II is indicated by an arrow.

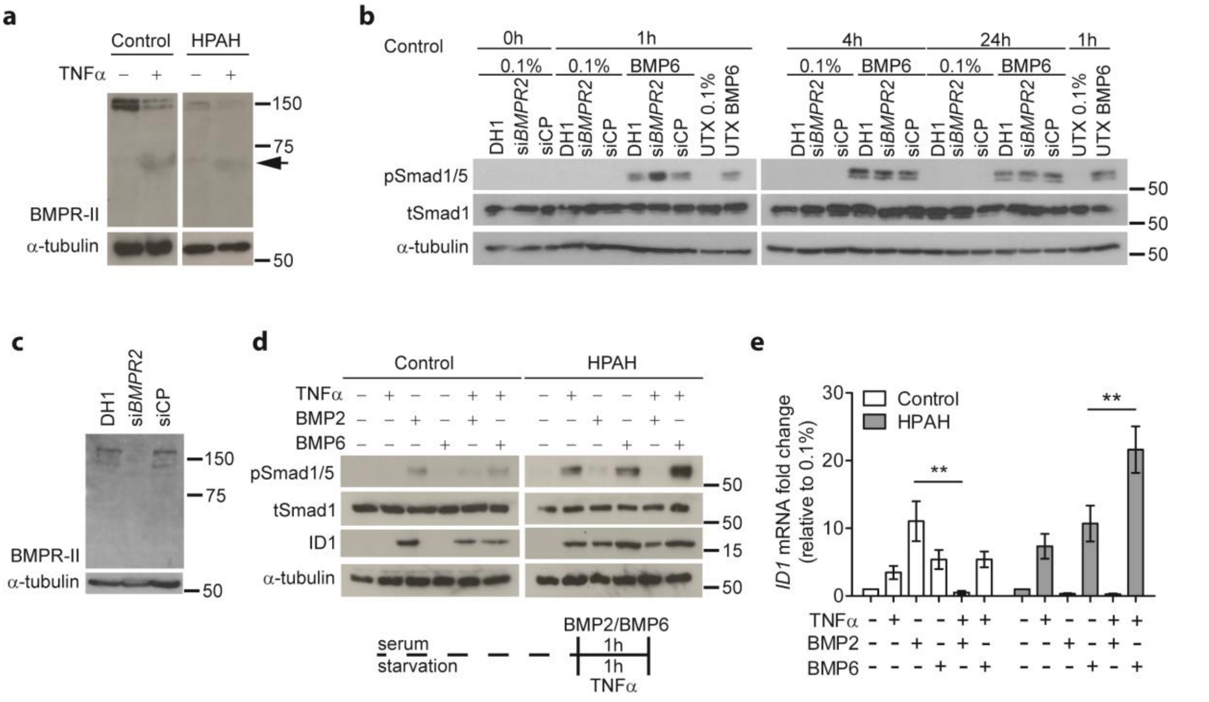


**Supplementary Figure 7. TNFα changes BMP6 signaling dynamics consistent with BMPR-II siRNA.** (**a**) Representative immunoblots of BMPR-II expression in human dPASMCs from disease-free controls and HPAH patients stimulated with TNFα (1 ng $mL^{-1}$) for 24 h. Blots were reprobed for α-tubulin to ensure equal loading. The data shown are representative of three control and HPAH cell lines. (**b** and **c**) Control human dPASMCs were transfected with *BMPR2* siRNA (si*BMPR2*) or control siRNA (siCP) using DharmaFECT1™ (DH1) followed by treatment with BMP6 (10 ng $mL^{-1}$) in 0.1% FBS for 1,4 or 24 hours. (**b**) Protein lysates were immunoblotted for phospho-Smad 1/5 and total Smad 1 followed by reprobing for α-tubulin to ensure equal loading. Data are representative of three separate experiments. (**c**) Immunoblotting for BMPR-II to confirm the loss of protein in siBMPR2-transfected PASMCs. Blots were reprobed for α-tubulin to ensure equal loading. Data are representative of three separate experiments. (**d**) Immunoblotting of phospho-Smad 1/5, total Smad 1 and ID1 expression in human dPASMCs from disease-free controls and HPAH patients co-stimulated with TNFα (1 ng $mL^{-1}$) and/or BMP2 or BMP6 (both 10 ng $mL^{-1}$) for 1 h. Blots were reprobed for α-tubulin to ensure equal loading. Data are representative of three control and HPAH cell lines. (**e**) *ID1* mRNA expression, normalized to *ACTB,* in human dPASMCs from disease-free controls and HPAH patients co-stimulated with TNFα (1 ng $mL^{-1}$) and/or BMP2 or BMP6 (both 10 ng $mL^{-1}$) for 1 h ($n$ = 3; one-way ANOVA with post-hoc Tukey's for multiple comparisons). ** $P \leq 0.01$. Error bars represent mean ± s.e.m. Lower molecular mass BMPR-II is indicated by an arrow.

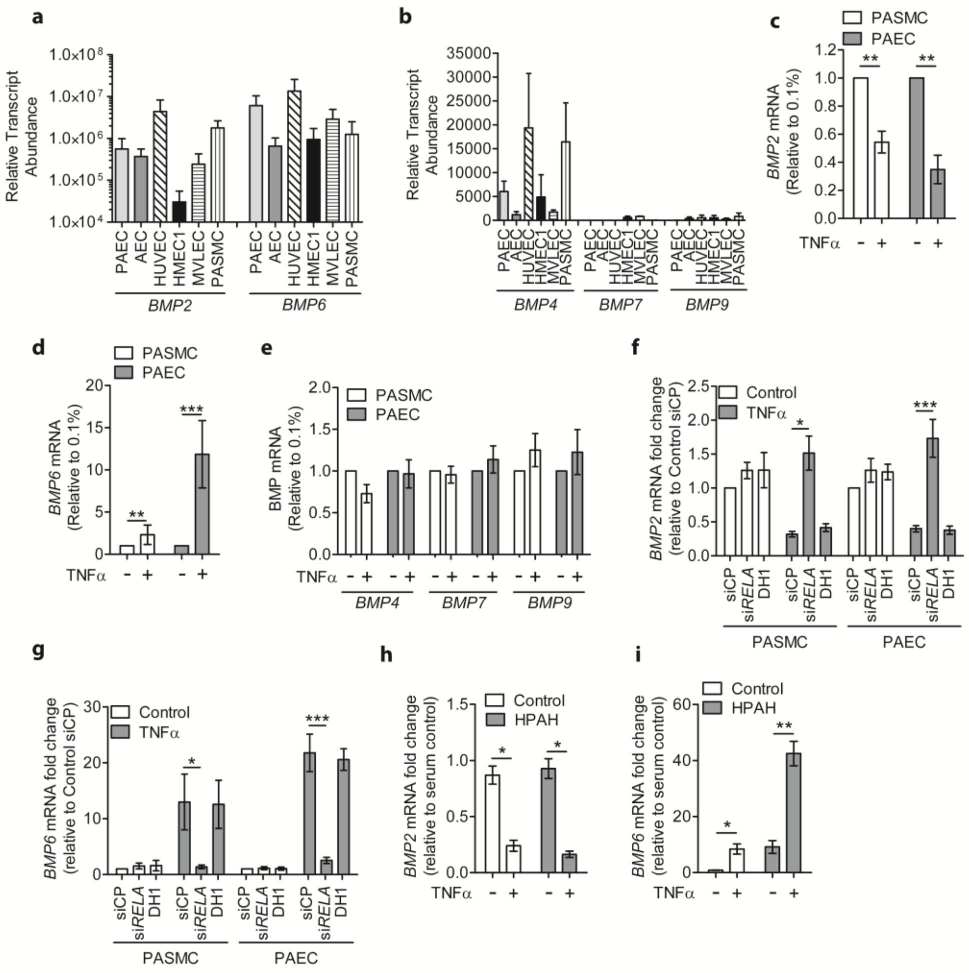


**Supplementary Figure 8. *BMP2* and *BMP6* are the most abundantly expressed ligands in vascular cells.** (**a** and **b**) Relative transcript abundance of *BMP2*, *BMP4*, *BMP6*, *BMP7* and *BMP9*, normalized to *B2M* and *ACTB* as detailed in the methods, in endothelial-derived cells (AECs, HMEC1s, HUVECs, HMVLECs, and PAECs) and distal smooth muscle cells (PASMCs) (n=3 independent experiments. (**c** - **e**) *BMP2* (**c**), *BMP6* (**d**), *BMP4*, *BMP7* and *BMP9* (**e**) mRNA expression, normalized to *ACTB*, in control human dPASMCs and PAECs treated with TNFα (1 ng $mL^{-1}$) for 24 h ($n$ = 5; Student's $t$-test). (**f** and **g**) *BMP2* and *BMP6* mRNA expression, normalized to *ACTB*, in control human dPASMCs and PAECs following treatment with DharmaFECT1$^{TM}$ alone (DH1), si*RELA* or non-targeting siRNA control (siCP) with or without 24 h TNFα (1 ng $mL^{-1}$) treatment ($n$ = 3; one-way ANOVA with post-hoc Tukey's for multiple comparisons). (**h** and **i**) *BMP2* and *BMP6* mRNA expression, normalized to *ACTB*, in human dPASMCs from disease-free controls and HPAH patients stimulated with or without TNFα (1 ng $mL^{-1}$) for 24 h ($n$ = 3; one-way ANOVA with post-hoc Tukey's for multiple comparisons). PAEC – human pulmonary artery endothelial cells; AEC - human aortic endothelial cells; HMEC1 - human microvascular endothelial cells; HUVEC – human umbilical vein endothelial cells; HMVLEC – human microvascular lung endothelial cells; PASMC – human distal pulmonary artery smooth muscle cells. *$P \leq 0.05$, **$P \leq 0.01$, ***$P \leq 0.001$. Error bars represent mean ± s.e.m.

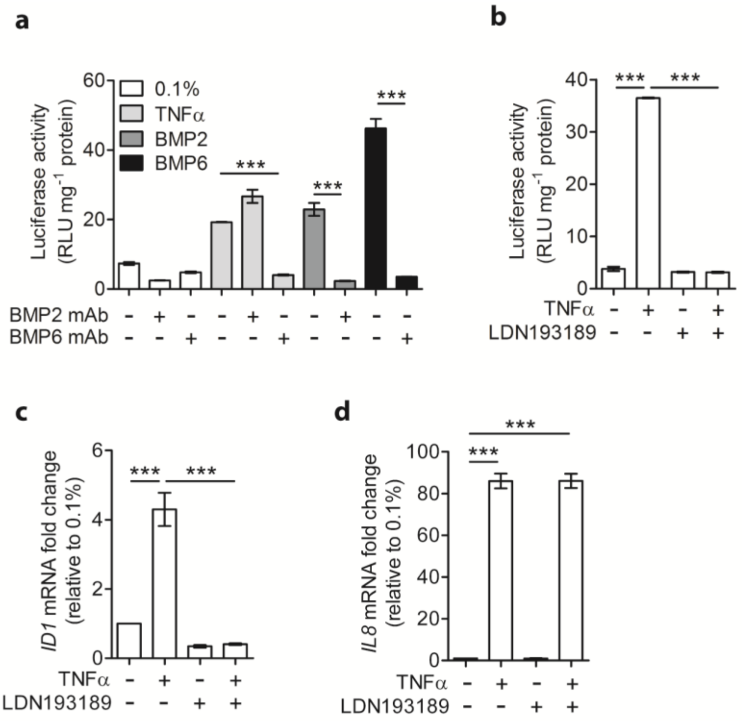


**Supplementary Figure 9. TNFα promotes BMP signalling via BMP6 induction.** (**a**) C2C12-BRE mouse myoblasts were treated with TNFα (1 ng mL$^{-1}$), BMP2 (10 ng mL$^{-1}$) or BMP6 (10 ng mL$^{-1}$) in the presence or absence of BMP2 mAb (1 µg mL$^{-1}$) or BMP6 mAb (1 µg mL$^{-1}$) for 24 h prior to measuring luciferase activity ($n$ = 3; repeated measures ANOVA with post-hoc Tukey's test). (**b**) C2C12-BRE mouse myoblasts were treated with TNFα (1 ng mL$^{-1}$) and LDN193189 (250 nM) for 16 h prior to measuring luciferase activity ($n$ = 3; repeated measures ANOVA with post-hoc Tukey's test). (**c** and **d**) *ID1* and *IL8* mRNA expression, normalized to *ACTB*, in control human dPASMCs treated with LDN193189 (250 nM) and/or TNFα (1 ng mL$^{-1}$) for 16 h as indicated. Data are expressed as the fold-change compared to the untreated control ($n$ = 5; one-way ANOVA with post-hoc Tukey's for multiple comparisons). ***$P \leq 0.001$. Error bars represent mean ± s.e.m.

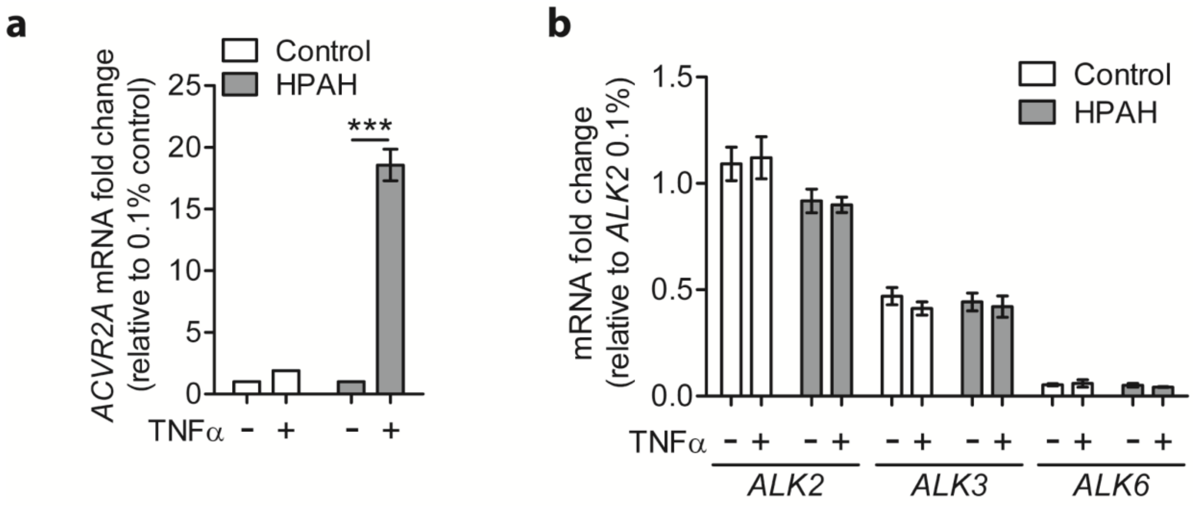


**Supplementary Figure 10. TNFα induces *ACVR2A* expression in HPAH PASMCs.** (**a**) *ACVR2A* mRNA expression, normalized to *ACTB*, in human dPASMCs from disease-free controls and HPAH patients stimulated with TNFα (1 ng $mL^{-1}$) for 24 h ($n$ = 3; Student's *t*-test). (**b**) *ALK2*, *ALK3* and *ALK6* mRNA expression, normalized to *ACTB*, in human dPASMCs from disease-free controls and HPAH patients stimulated with TNFα (1 ng $mL^{-1}$) for 24 h. ***$P \leq 0.001$. Error bars represent mean $\pm$ s.e.m.

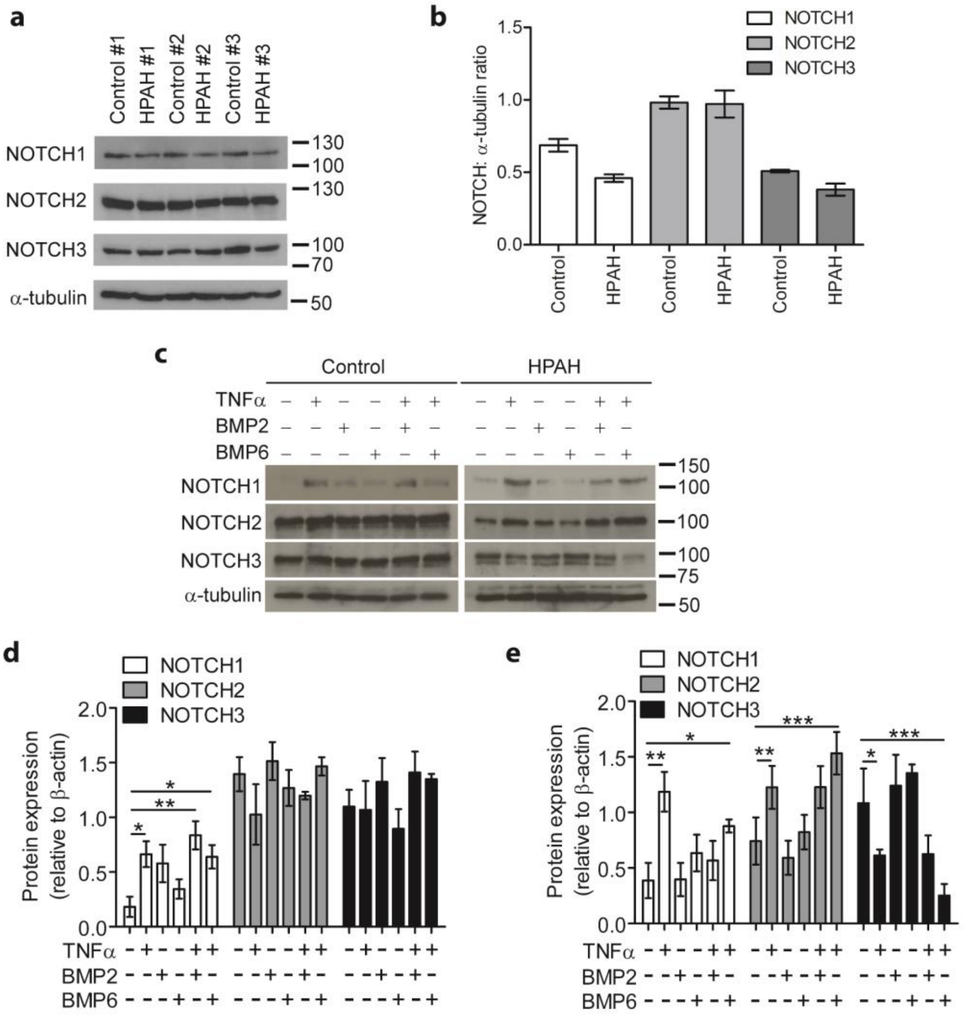


**Supplementary Figure 11. TNFα selectively alters NOTCH expression in HPAH PASMCs.** (**a**) Immunoblotting for cleaved/transmembrane intracellular NTM (NTM) regions for NOTCH1, NOTCH2 and NOTCH3 expression in control disease-free and HPAH dPASMCs (n=3 different lines for each). Blots were reprobed for α-tubulin to ensure equal loading. (**b**) Densitometry of NTM regions from blots in panel **a** relative to α-tubulin levels. (**c**) Immunoblotting for NTM regions for NOTCH1, NOTCH2 and NOTCH3 expression in control disease-free and HPAH dPASMCs treated with TNFα (1 ng $mL^{-1}$) and/or BMP2 or BMP6 (both 10 ng $mL^{-1}$) for 1 h as indicated. Blots were reprobed for α-tubulin to ensure equal loading. (**d** and **e**) Densitometry of NTM regions for immunoblots in panel **c** relative to α-tubulin levels. ($n$ = 3; Mann-Whitney Test). *$P \leq 0.05$, **$P \leq 0.01$, ***$P \leq 0.001$. Error bars represent mean ± s.e.m.

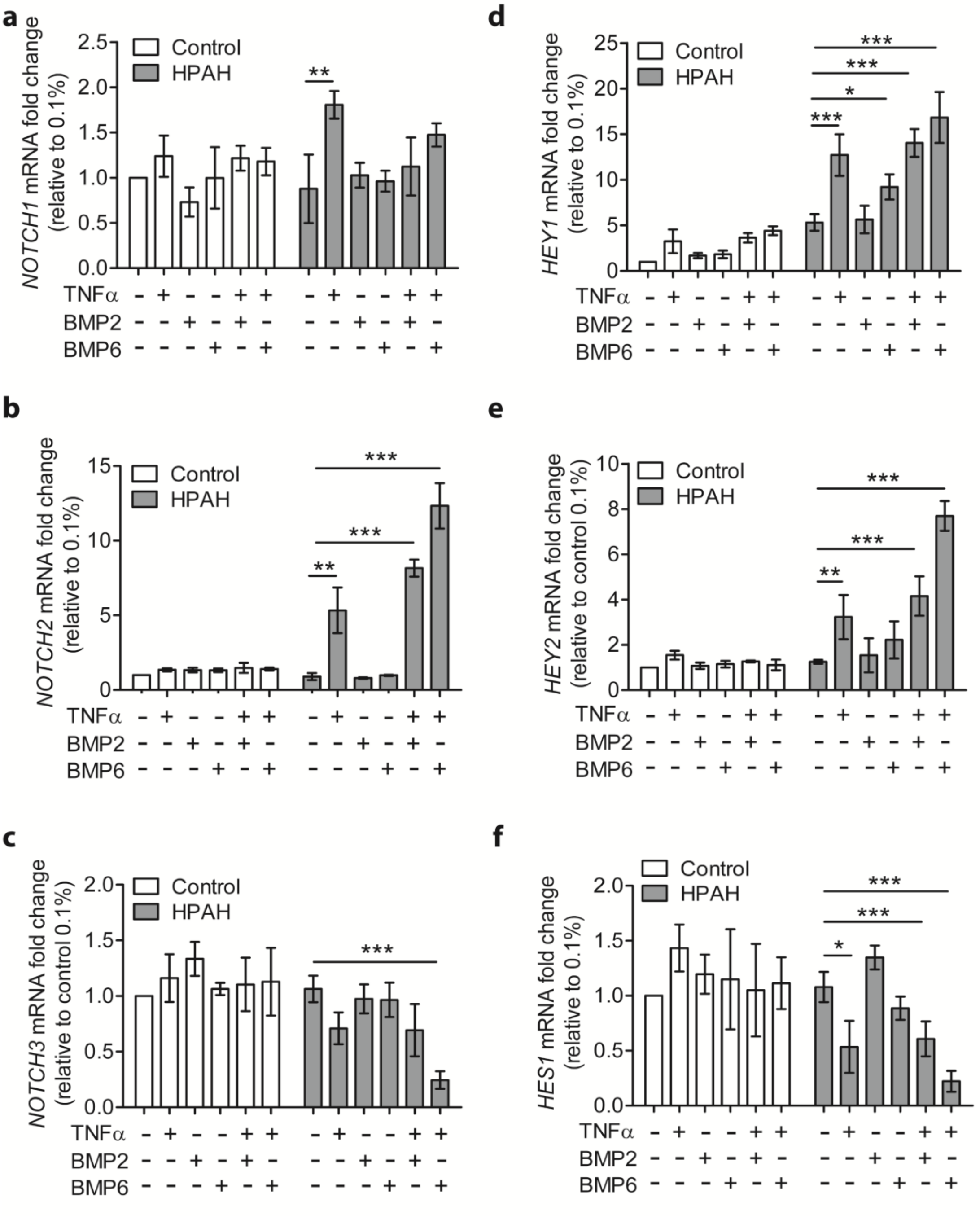


**Supplementary Figure 12. *NOTCH* and its targets are altered by TNFα in HPAH PASMCs.** (**a**-**f**) *NOTCH1* (**a**), *NOTCH2* (**b**), *NOTCH3* (**c**), *HEY1* (**d**), *HEY2* (**e**) and *HES1* (**f**) mRNA expression, normalized to *ACTB*, in human dPASMCs from disease-free controls and HPAH patients stimulated with TNFα (1 ng $mL^{-1}$) and/or BMP2 or BMP6 (both 10 ng $mL^{-1}$) for 1 h as indicated. One-way ANOVA with post-hoc Tukey's for multiple comparisons used in **a**, **b**, **c**, **d**, **e** and **f**. $^{*}P \leq 0.05$, $^{**}P \leq 0.01$, $^{***}P \leq 0.001$. Error bars represent mean ± s.e.m.

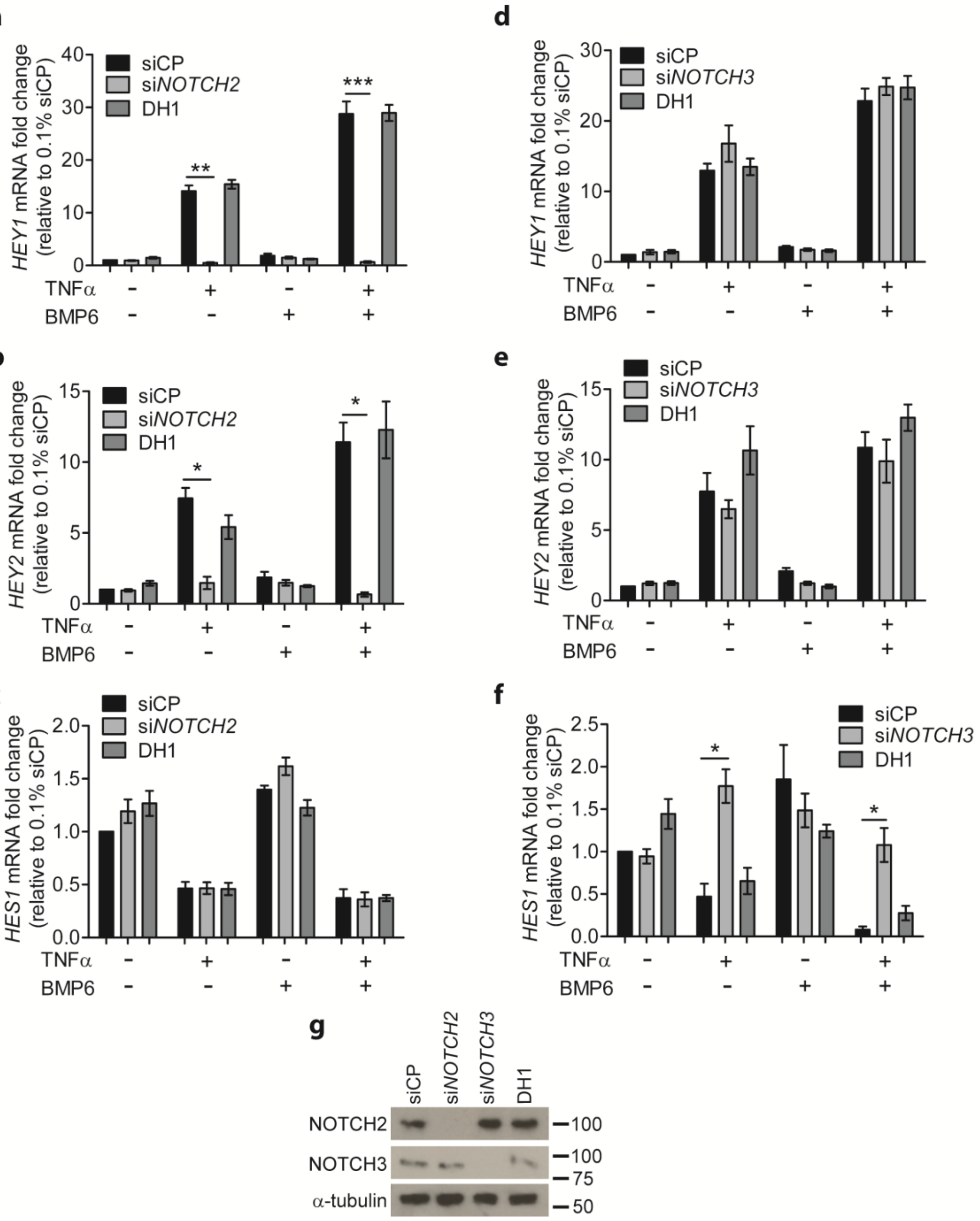


**Supplementary Figure 13. *HEY1* and *HEY2* are targets of *NOTCH2*; *HES1* is a target of *NOTCH3*.** (**a** - **c**) *HEY1* (**a**), *HEY2* (**b**) and *HES1* (**c**) mRNA expression, normalized to *ACTB*, in human HPAH dPASMCs transfected with DharmaFECT1$^{TM}$ alone (DH1), si*NOTCH2* or non-targeting siRNA control (siCP) and followed by treatment with TNFα (1 ng mL) and/or BMP6 (10 ng $mL^{-1}$) for 1 h as indicated ($n$ = 3). (**d** - **f**) *HEY1* (**d**), *HEY2* (**e**) and *HES1* (**f**) mRNA expression, normalized to *ACTB*, in human HPAH dPASMCs transfected with DharmaFECT1$^{TM}$ alone (DH1), si*NOTCH3* or non-targeting siRNA control (siCP) followed by treatment with TNFα (1 ng $mL^{-1}$) and/or BMP6 (10 ng $mL^{-1}$) for 1 h as indicated ($n$ = 3). (**g**) Immunoblot of NOTCH2 and NOTCH3 cleaved/transmembrane intracellular (NTM) regions in human HPAH dPASMCs transfected with DH1 alone , si*NOTCH2*, si*NOTCH3* or siCP . One-way ANOVA with post-hoc Tukey's for multiple comparisons used in **a**, **b** and **f**. $*P \leq 0.05$, $**P \leq 0.01$, $***P \leq 0.001$. Error bars represent mean ± s.e.m.

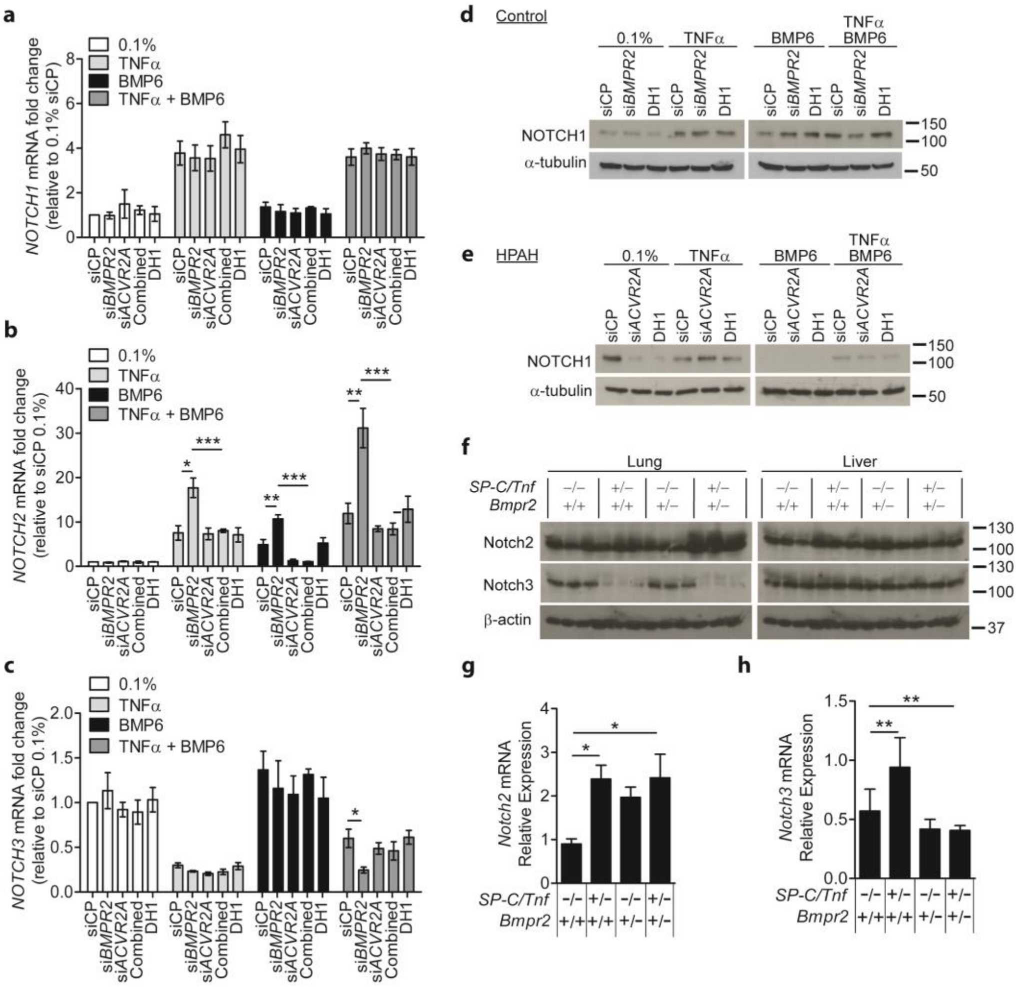


**Supplementary Figure 14. TNFα alters *NOTCH* expression differentially in *BMPR2* deficiency.** (**a** - **c**) *NOTCH1* (**a**), *NOTCH2* (**b**) and *NOTCH3* (**c**) mRNA expression, normalized to *ACTB*, in human control dPASMCs transfected with DharmaFECT1™ alone (DH1), si*BMPR2*, si*ACVR2A*, combined si*BMPR2* + si*ACVR2A* or non-targeting siRNA control (siCP) followed by treatment with TNFα (1 ng $mL^{-1}$) and/or BMP6 (10 ng $mL^{-1}$) for 1 h as indicated (*n* = 3; *n* = 4 for NOTCH3). (**d** and **e**) Representative immunoblots of NOTCH1 NTM region in (**d**) human disease-free control PASMCs following transfection with DH1 Alone, si*BMPR2*, or siCP or (**e**) HPAH dPASMCs following transfection with DH1 alone , si*ACVR2A*, or siCP followed by treatment with TNFα (1 ng $mL^{-1}$) and/or BMP6 (10 ng mL) for 1 h as indicated. Reprobed for α-tubulin to ensure equal loading. The data shown are representative of three experiments. Note that a consistent increase of NOTCH1 NTM region was observed with siCP transfection in HPAH PASMCs at baseline. (**f**) Immunoblots of Notch2 and Notch3 cleaved/transmembrane intracellular (NTM) regions for expression in lungs and livers isolated from 8-9 week old *Bmpr2*$^{+/+}$, *SP-C/Tnf/Bmpr2*$^{+/+}$, *Bmpr2*$^{+/-}$ and *SP-C/Tnf/Bmpr2*$^{+/-}$ transgenic mice (*n* = 3 per group). Blots were reprobed for β-actin to ensure equal loading. (**g** and **h**) *Notch2* and *Notch3* mRNA expression, normalized to *Actb*, in livers isolated from 8-9 week old *Bmpr2*$^{+/+}$, *SP-C/Tnf/Bmpr2*$^{+/+}$, *Bmpr2*$^{+/-}$ and *SP-C/Tnf/Bmpr2*$^{+/-}$ transgenic mice (*n* = 4). One-way ANOVA with post-hoc Tukey's for multiple comparisons used in **a**, **b**, **c**, **g** and **h**. $*P \leq 0.05$, $**P \leq 0.01$, $***P \leq 0.001$. Error bars represent mean ± s.e.m.

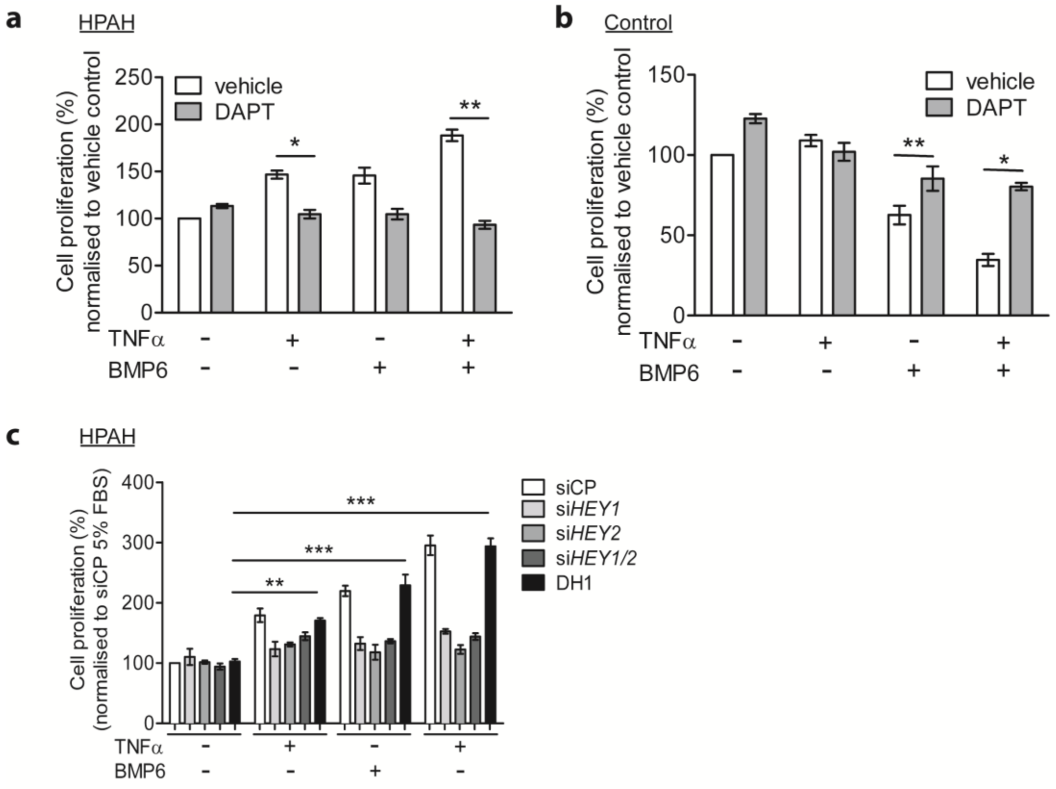


**Supplementary Figure 15. DAPT inhibits TNFα influence on PASMC proliferation**. (**a** and **b**) Day 6 assessment of human HPAH (**a**) and control (**b**) dPASMCs proliferation following pre-treatment with DAPT (5 µM) for 30 min before subsequent TNFα (1 ng $mL^{-1}$) and/or BMP6 (50 ng $mL^{-1}$) treatment for 24 h ($n$ = 3; Student's *t*-test). (**c**) Proliferation of human HPAH dPASMCs on day 6 following transfection with DharmaFECT1$^{TM}$ alone (DH1), si*HEY1*, si*HEY2*, si*HEY1*+si*HEY2* (si*HEY1/2*) or non-targeting siRNA control (siCP) and treatment every 48 h with TNFα (1 ng $mL^{-1}$) and/or BMP6 (50 ng $mL^{-1}$) as indicated ($n$ = 3; one-way ANOVA with post-hoc Tukey's for multiple comparisons). $^{*}P \leq 0.05$, $^{**}P \leq 0.01$, $^{***}P \leq 0.001$. Error bars represent mean $\pm$ s.e.m.

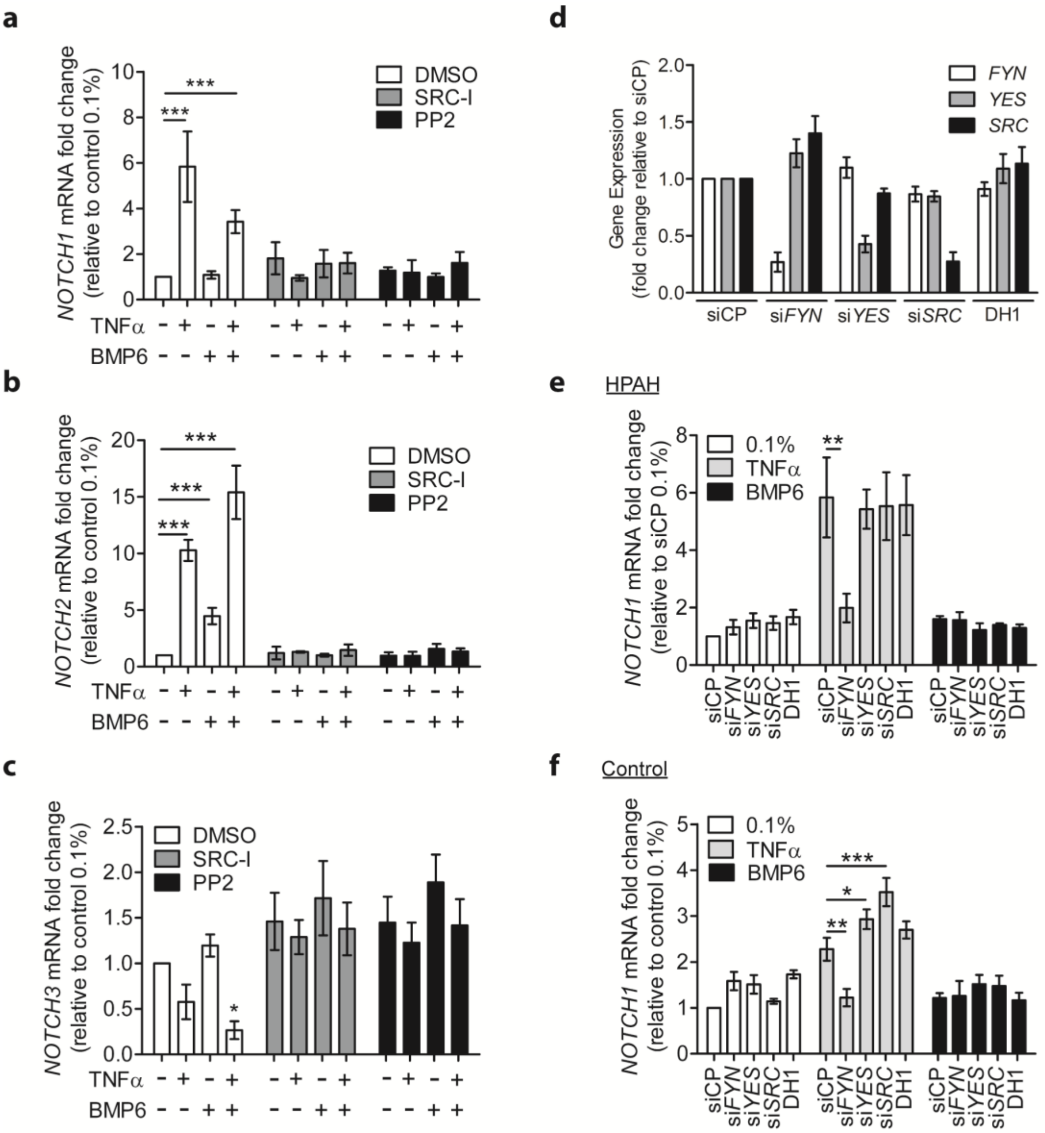


**Supplementary Figure 16. Inhibition of SRC kinases reverses TNFα and BMP6 regulation of *NOTCH*.** (**a** - **c**) *NOTCH1* (**a**), *NOTCH2* (**b**) and *NOTCH3* (**c**) mRNA expression, normalized to *ACTB*, in human HPAH dPASMCs pre-incubated with SRC-I (1µM) or PP2 (250 nM) in DMSO for 1 h before stimulation with TNFα (1 ng $mL^{-1}$) and/or BMP6 (10 ng $mL^{-1}$) for 1 h as indicated (*n* = 3; one-way ANOVA with post-hoc Tukey's for multiple comparisons). (**d**) Human *FYN*, *YES*, and *SRC* mRNA expression in control dPASMCs following transfection with DharmaFECT1$^{TM}$ alone (DH1), si*FYN*, si*YES*, si*SRC* or non-targeting siRNA control (siCP). (**e** and **f**) *NOTCH1* mRNA expression, normalized to *ACTB*, in control (**e**) and HPAH (**f**) human dPASMCs following transfection with DH1 alone, si*FYN*, si*YES*, si*SRC* or siCP and treated with TNFα (1 ng $mL^{-1}$) and/or BMP6 (10 ng $mL^{-1}$) for 1 h as indicated (*n* = 3; one-way ANOVA with post-hoc Tukey's for multiple comparisons). $*P \leq 0.05$, $**P \leq 0.01$, $***P \leq 0.001$. Error bars represent mean ± s.e.m.

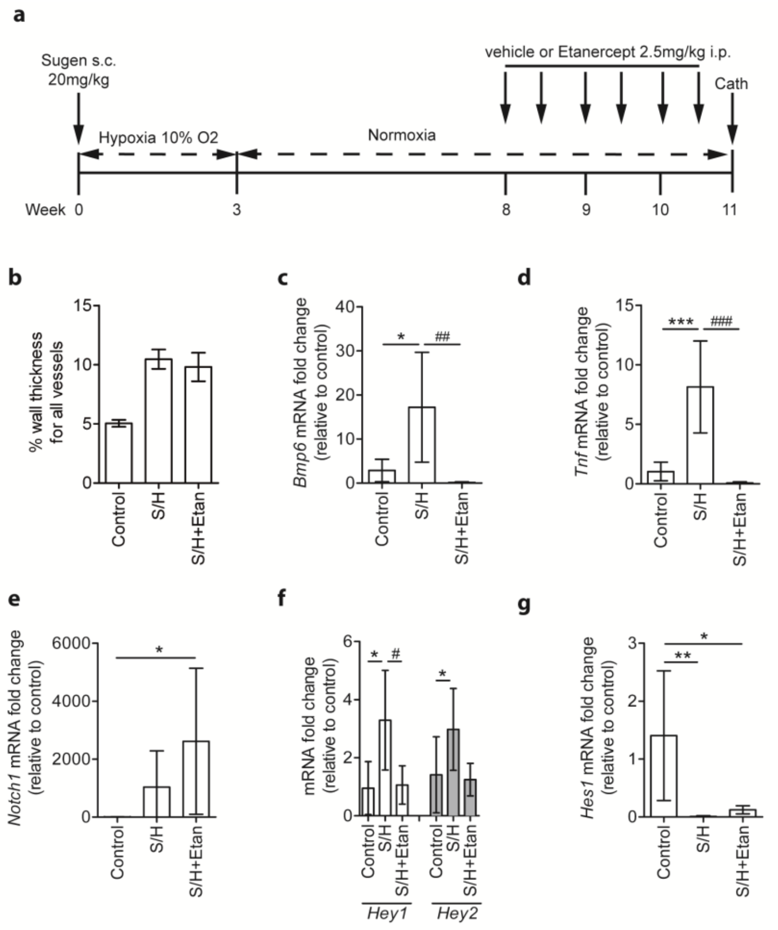


**Supplementary Figure 17. Etanercept reverses established pulmonary hypertension in the Sugen-hypoxia model.** (**a**) Rats were given vehicle injections and maintained in normoxia (Control, *n* = 6) or challenged with SU-5416 (20 mg/kg, s.c.) and 3 weeks of hypoxia (10% O2) before 5 weeks of normoxia and 3 weeks of biweekly treatment with saline vehicle (S/H, *n* = 9) or etanercept (S/H+Etan, *n* = 9; 2.5 mg/kg, i.p.). (**b**) Assessment of pulmonary arterial wall thickness as a percentage of luminal diameter. (**c - g**) *Bmp6* **(c)**, *Tnf* **(d)**, *Notch1* **(e)**, *Hey1* and *Hey2* **(f)** and *Hes1* **(g)** mRNA expression, normalized to *Actb*, in lungs isolated from control, S/H and S/H+Etan rats (*n* = 6). One-way ANOVA with post-hoc Tukey's for multiple comparisons. S/H = Sugen-hypoxia, S/H+Etan = Sugen-hypoxia + Etanercept */#$P \leq 0.05$, **/##$P \leq 0.01$, ***/###$P \leq 0.001$. Error bars represent mean $\pm$ s.e.m.

a) Figure 1c_BMPR-II (left panel)

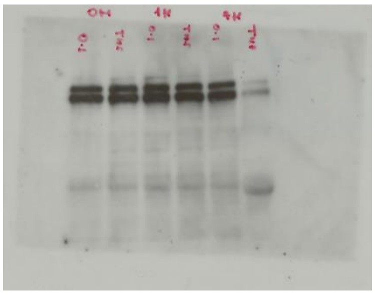

b) Figure 1c_alpha-tubulin (left panel)

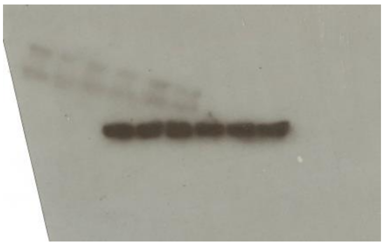

c) Figure 1c_BMPR-II (right panel)

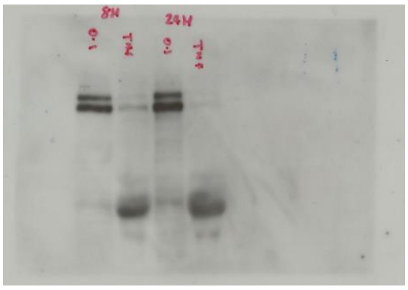

d) Figure 1c_alpha-tubulin (right panel)

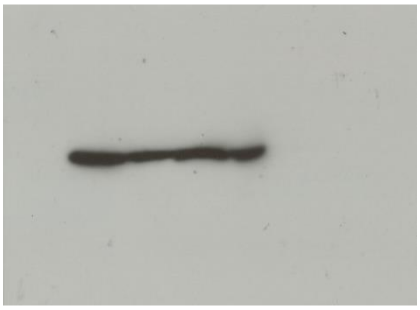

e) Figure 1d_BMPR-II

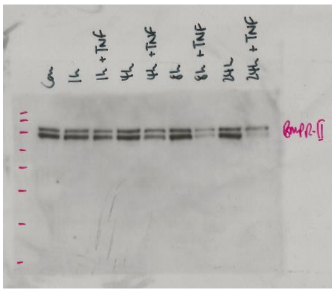

f) Figure 1d_alpha-tubulin

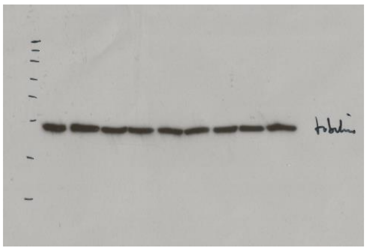

g) Figure 1g_BMPR-II

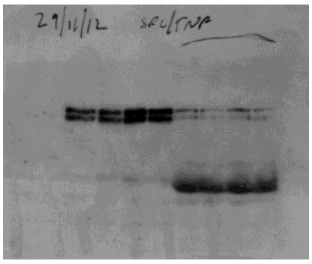

h) Figure 1g_alpha tubulin

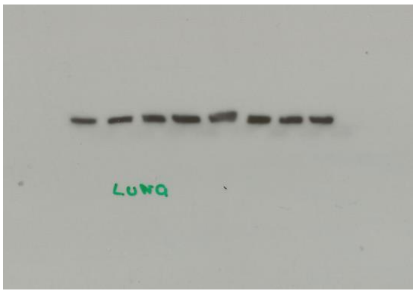

**Supplementary Figure 18. Uncropped western blots for Figures 1c-g.** The relevant figures are indicated in the blot titles.

a) Figure 1h_BMPR-II (left panel)

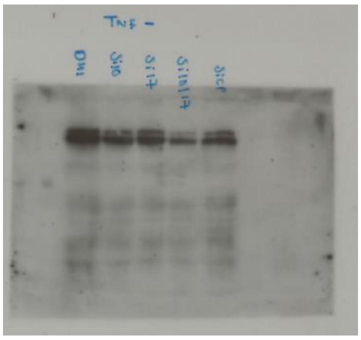

e) Figure 1h_BMPR-II (right panel)

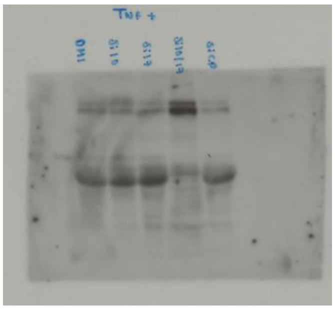

b) Figure 1h_ADAM10 (left panel)

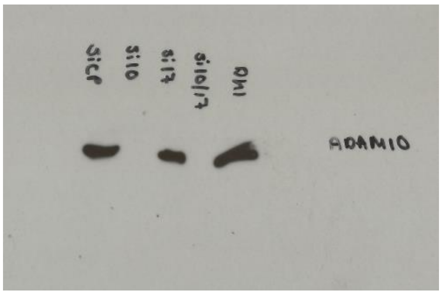


f) Figure 1h_ADAM10 (right panel)

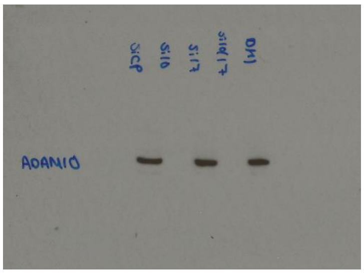


c) Figure 1h_ADAM17 (left panel)

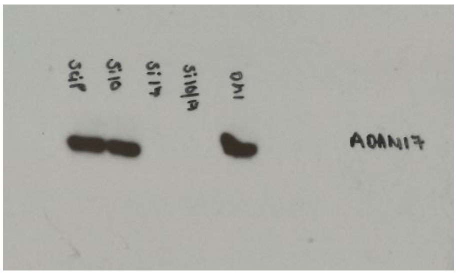


g) Figure 1h_ADAM17 (right panel)

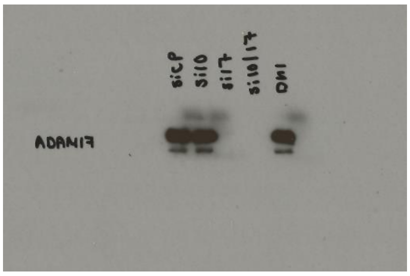


d) Figure 1h_alpha tubulin (left panel)

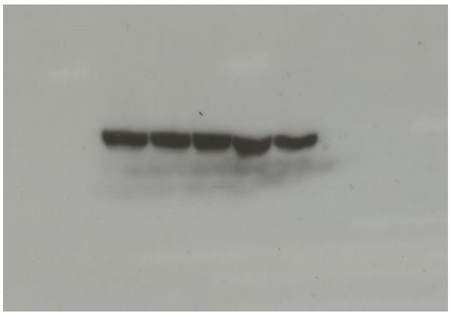

h) Figure 1h_alpha tubulin (right panel)

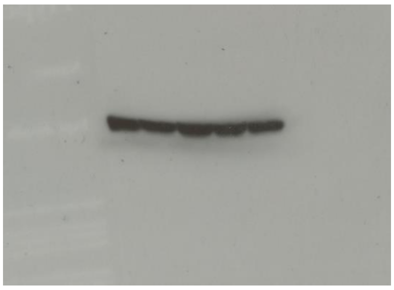

**Supplementary Figure 19. Uncropped western blots for Figure 1h.** The relevant figures are indicated in the blot titles.

a) Figure 2b_pSMAD1/5 (left panel)

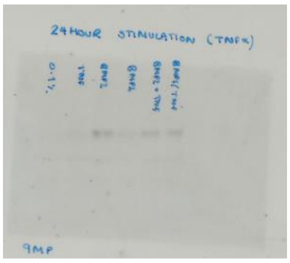


b) Figure 2b_tSMAD1 (left panel)

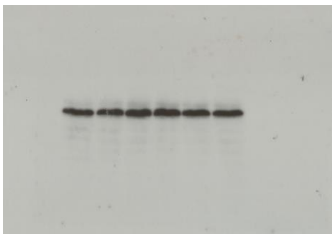

c) Figure 2b_ID1 (left panel)

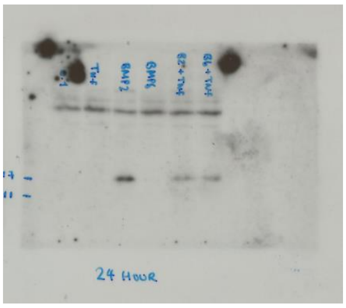


d) Figure 2b_alpha tubulin (left panel)

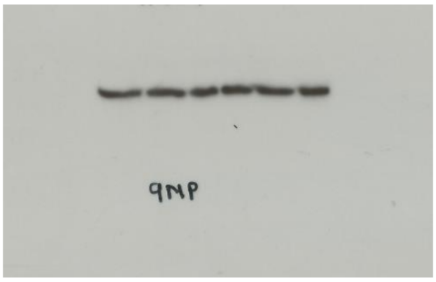


e) Figure 2b_pSMAD1/5 (right panel)

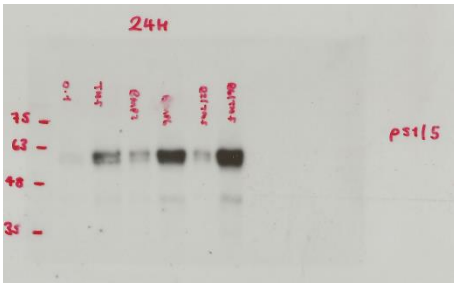


f) Figure 2b_tSMAD1 (right panel)

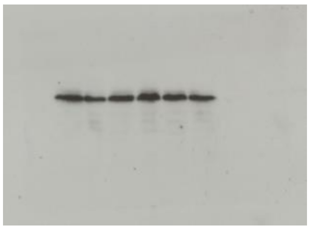

g) Figure 2b_ID1 (right panel)

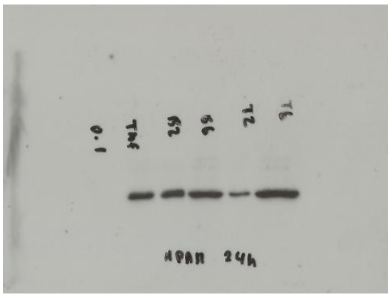

h) Figure 2b_alpha tubulin (right panel)

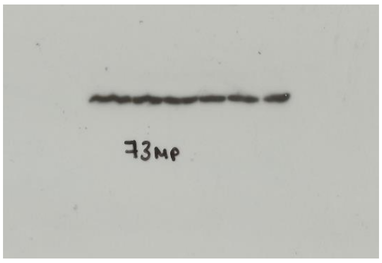


**Supplementary Figure 20. Uncropped western blots for Figure 2b**. The relevant figures are indicated in the blot titles.

a) Figure 3e_pSMAD1/5 (left panel)

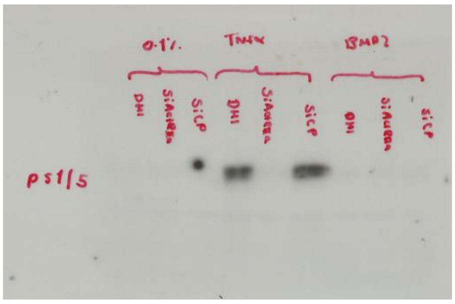

b) Figure 3e_tSMAD1 (left panel)

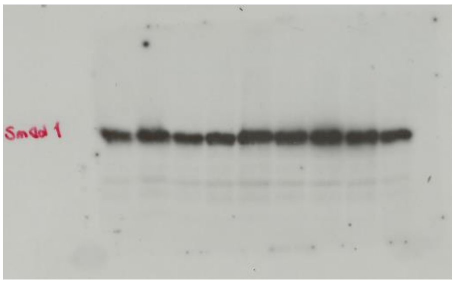

c) Figure 3e_ID1 (left panel)

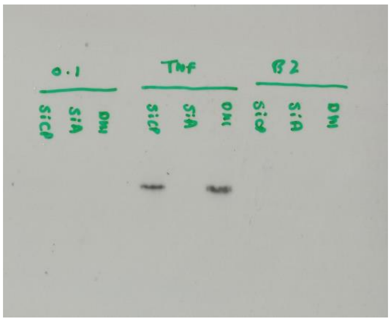

d) Figure 3e_alpha tubulin (left panel)

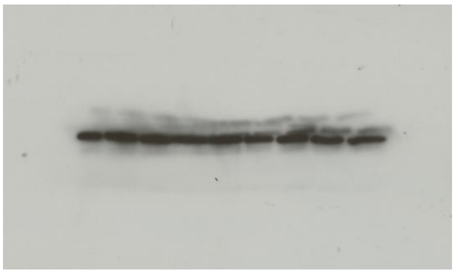

e) Figure 3e_pSMAD1/5 (right panel)

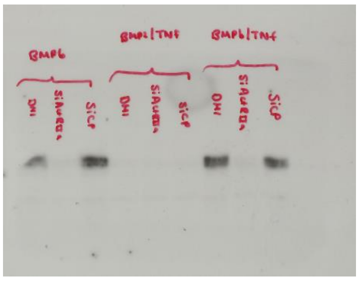

f) Figure 3e_tSMAD1 (right panel)

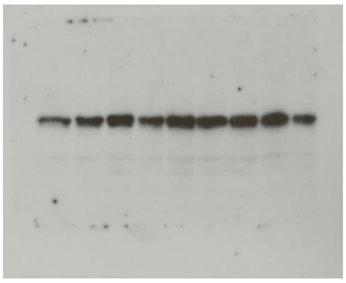

g) Figure 3e_ID1 (right panel)

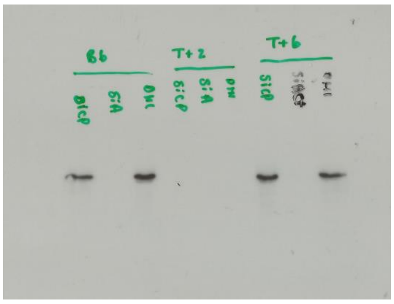

h) Figure 3e_alpha tubulin (right panel)

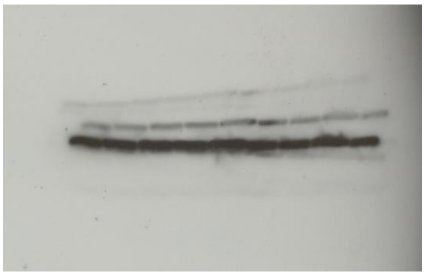

**Supplementary Figure 21. Uncropped western blots for Figure 3e**. The relevant figures are indicated in the blot titles.

a) Figure 4a_NOTCH2 (left panel)

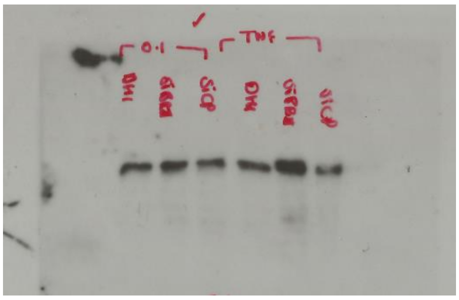


d) Figure 4a_NOTCH2 (right panel)

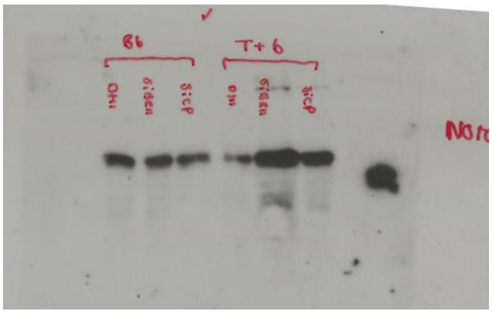


b) Figure 4a_NOTCH3 (left panel)

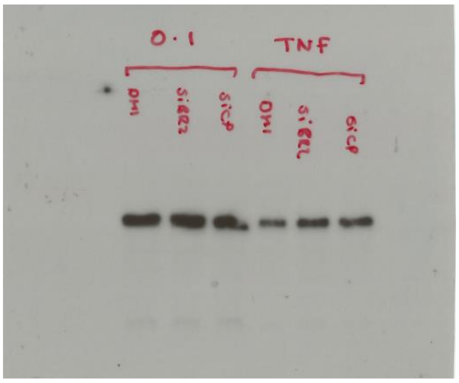


e) Figure 4a_NOTCH3 (right panel)

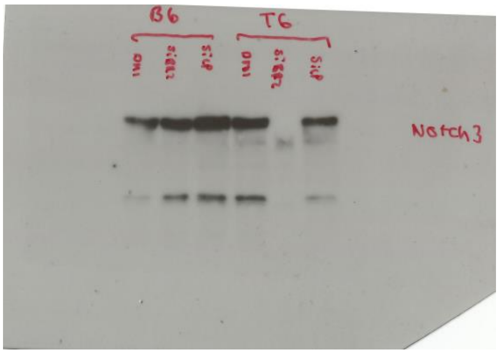


c) Figure 4a_alpha tubulin (left panel)

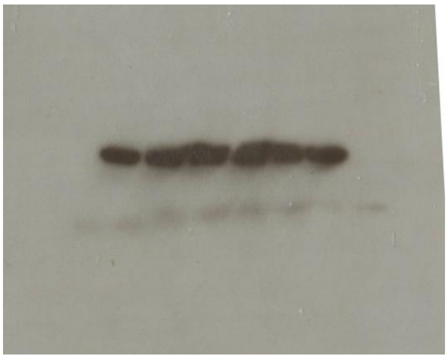

f) Figure 4a_alpha tubulin (right panel)

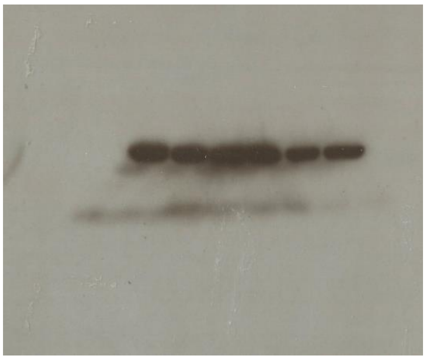

**Supplementary Figure 22. Uncropped western blots for Figure 4a**. The relevant figures are indicated in the blot titles.

a) Figure 4b_NOTCH2 (left panel)

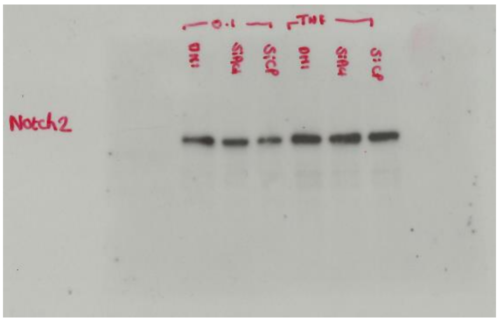


d) Figure 4b_NOTCH2 (right panel)

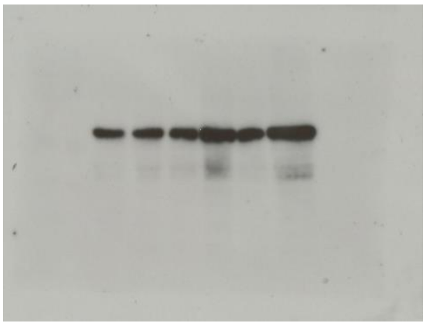

b) Figure 4b_NOTCH3 (left panel)

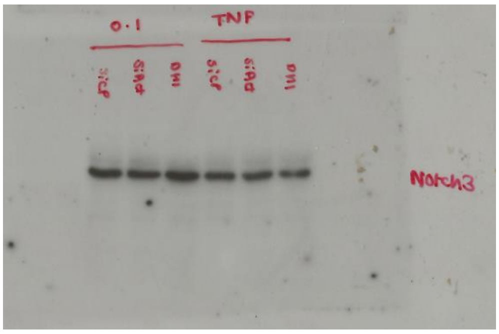


e) Figure 4b_NOTCH3 (right panel)

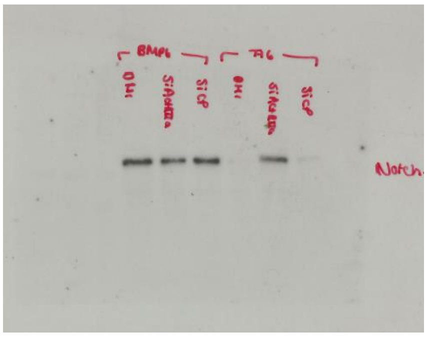

c) Figure 4b_alpha tubulin (left panel)

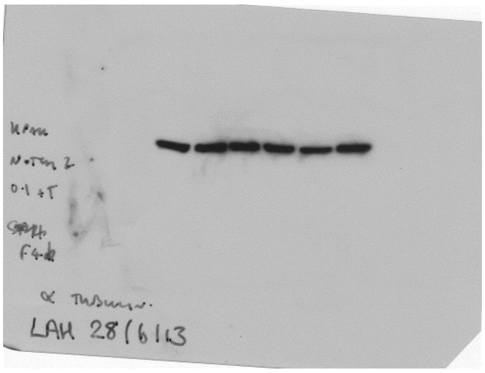


f) Figure 4b_alpha tubulin (right panel)

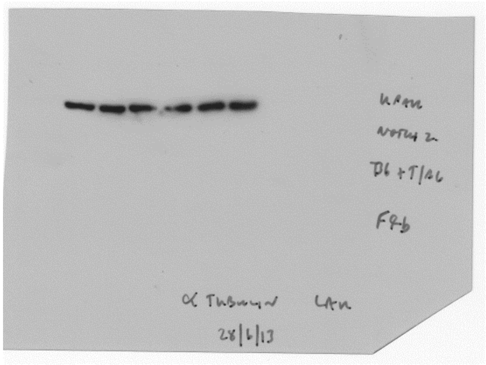


**Supplementary Figure 23. Uncropped western blots for Figure 4b.** The relevant figures are indicated in the blot titles.

a) Figure 5b_pSRC(527) (left panel)

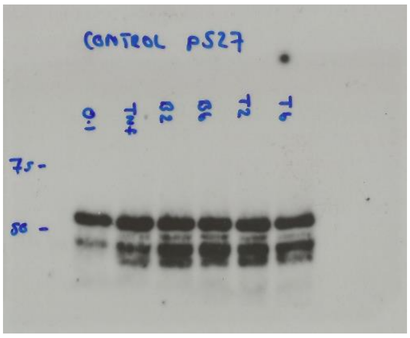

e) Figure 5b_pSRC(527) (right panel)

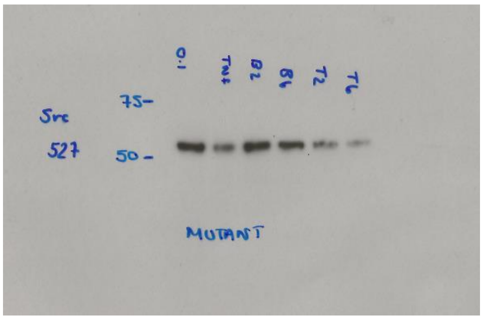

b) Figure 5b_pSRC(416) (left panel)

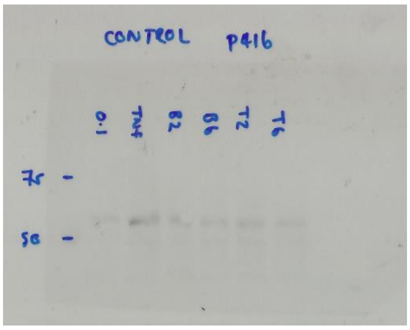

f) Figure 5b_pSRC(416) (right panel)

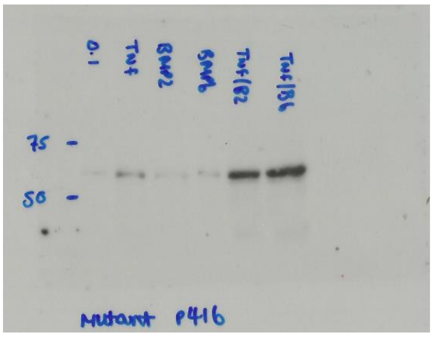

c) Figure 5b_SRC (left panel)

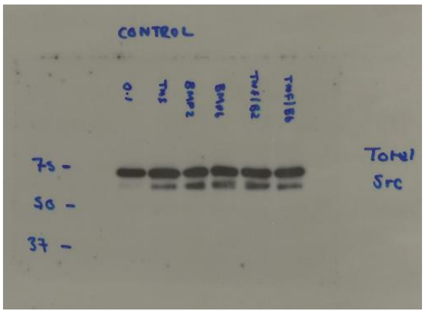

g) Figure 5b_SRC (right panel)

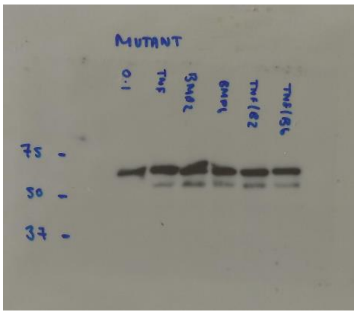

d) Figure 5b_alpha tubulin (left panel)

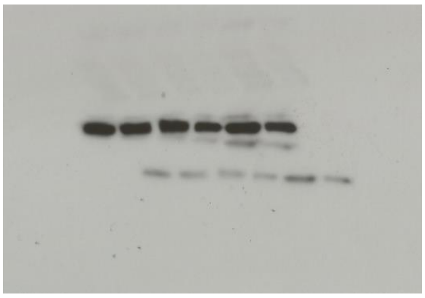

h) Figure 5b_alpha tubulin (right panel)

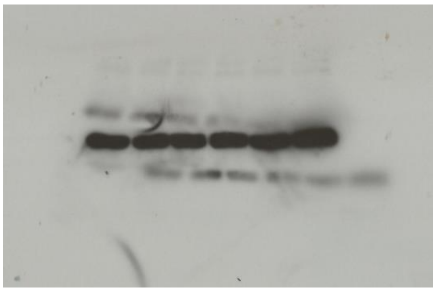

**Supplementary Figure 24. Uncropped western blots for Figure 5b.** The relevant figures are indicated in the blot titles.

a) Figure 5c_pSRC(527) (left panel)

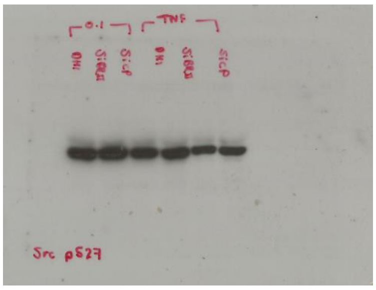


e) Figure 5c_pSRC(527) (right panel)

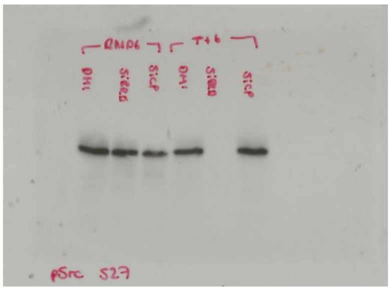


b) Figure 5c_pSRC(416) (left panel)

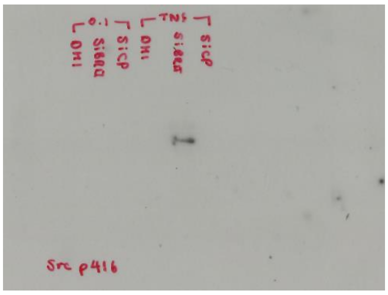


f) Figure 5c_pSRC(416) (right panel)

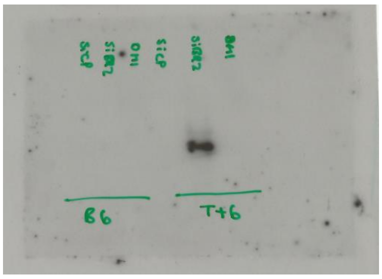


c) Figure 5c_SRC (left panel)

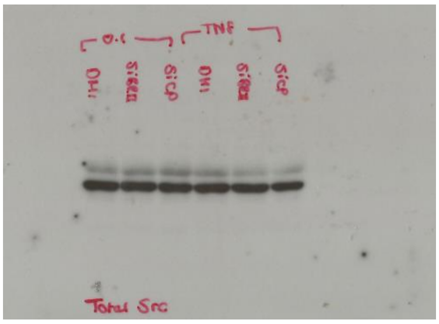


g) Figure 5c_SRC (right panel)

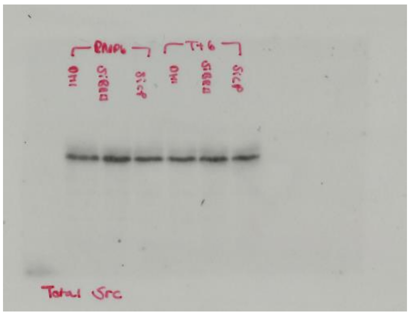


d) Figure 5c_alpha tubulin (left panel)

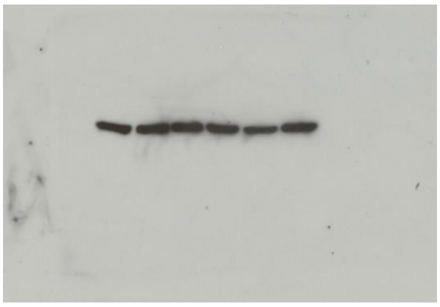

h) Figure 5c_alpha tubulin (right panel)

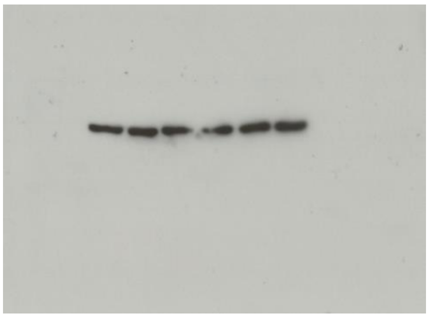

**Supplementary Figure 25. Uncropped western blots for Figure 5c.** The relevant figures are indicated in the blot titles.

a) Figure 5d_pSRC(527) (left panel)

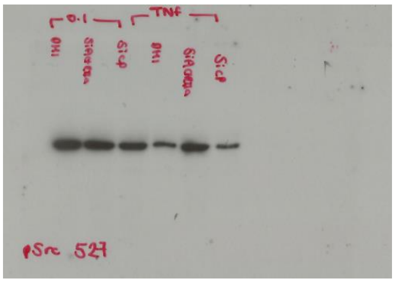


b) Figure 5d_pSRC(416) (left panel)

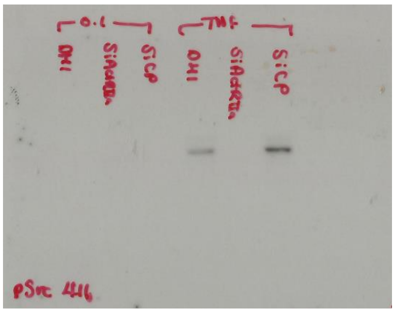


c) Figure 5d_SRC (left panel)

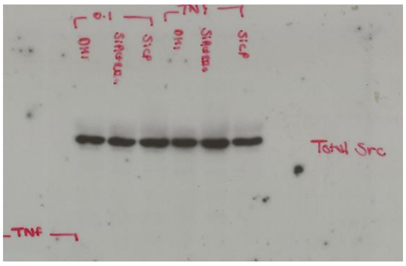


d) Figure 5d_alpha tubulin (left panel)

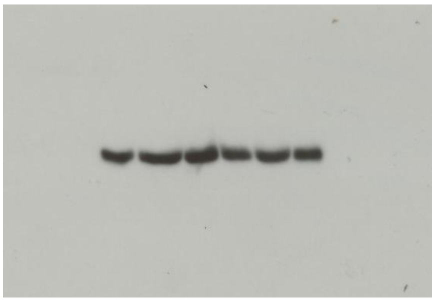

e) Figure 5d_pSRC(527) (right panel)

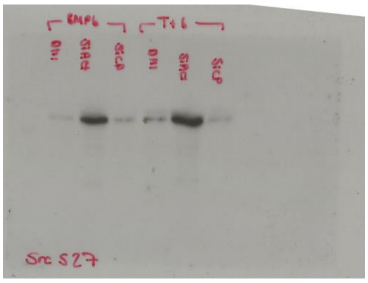

f) Figure 5d_pSRC(416) (right panel)

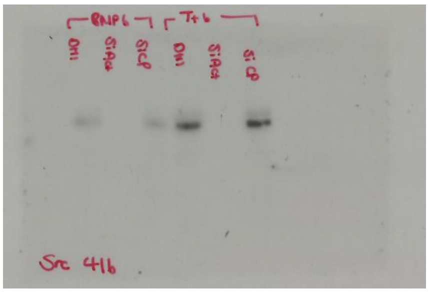


g) Figure 5d_SRC (right panel)

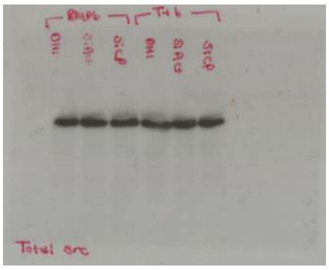

h) Figure 5d_alpha tubulin (right panel)

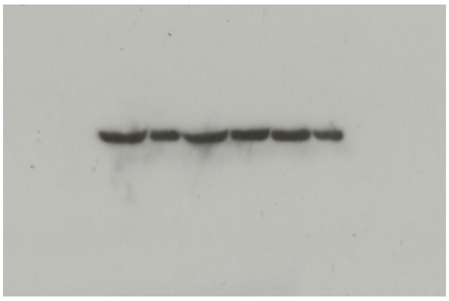

**Supplementary Figure 26. Uncropped western blots for Figure 5d**. The relevant figures are indicated in the blot titles.

a) Figure 6e_BMPR-II

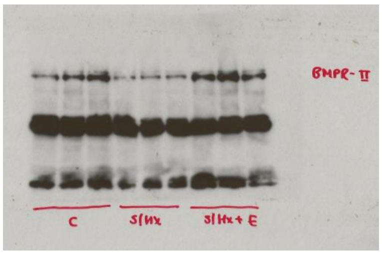

b) Figure 6e_pSmad1/5

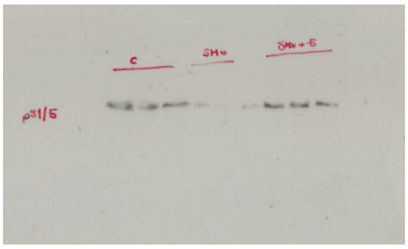

c) Figure 6e_Smad1

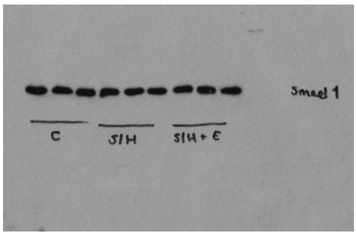

d) Figure 6e_Notch2

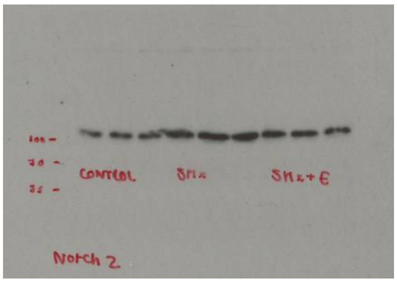

e) Figure 6e_Notch3

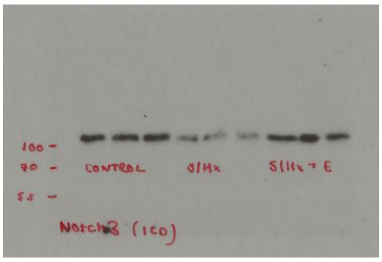

f) Figure 6e_Cleaved caspase-3

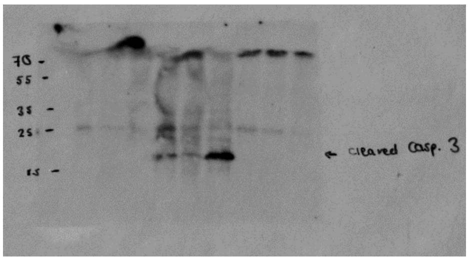

g) Figure 6e_caspase-3

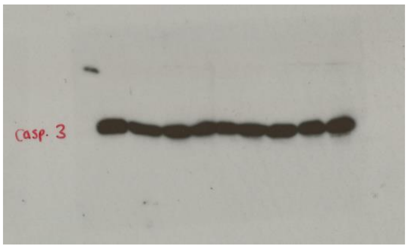

h) Figure 6e_alpha-SMA

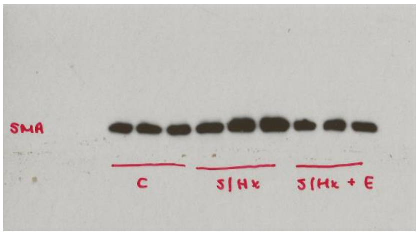

i) Figure 6e_alpha tubulin

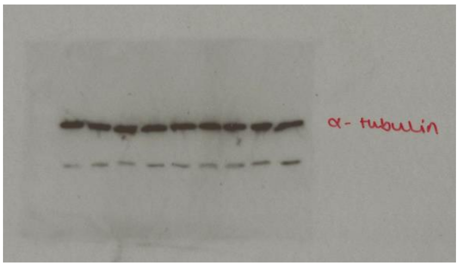

**Supplementary Figure 27. Uncropped western blots for Figure 6e**. The relevant figures are indicated in the blot titles.

a) Supplement Fig 1a_BMPR-II

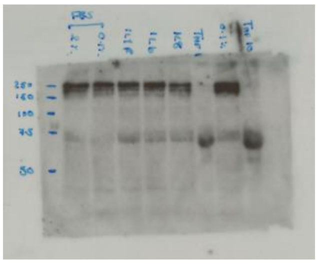

b) Supplement Fig 1a_alpha tubulin

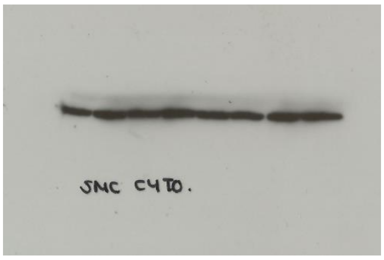


c) Supplement Fig 1b_BMPR-II

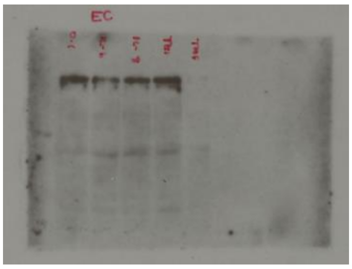


d) Supplement Fig 1b_alpha tubulin

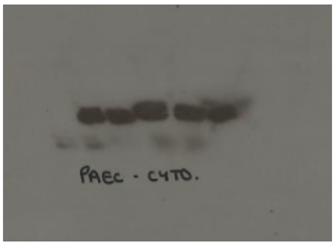


e) Supplement Fig 2a_BMPR-II

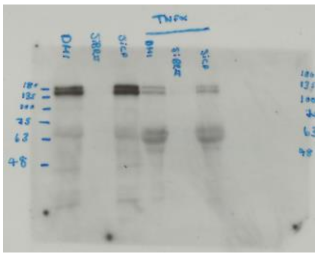

f) Supplement Fig 2a_alpha tubulin

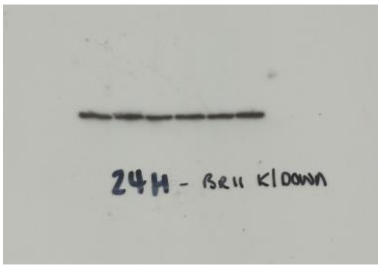

g) Supplement Fig 2b_BMPR-II

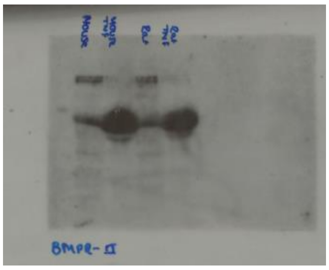


h) Supplement Fig 2b_alpha tubulin

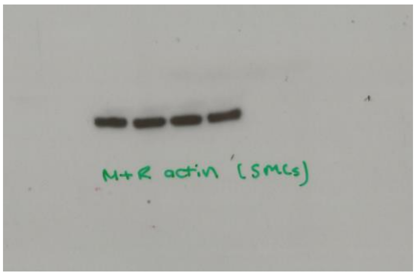


i) Supplement Fig 2c_BMPR-II

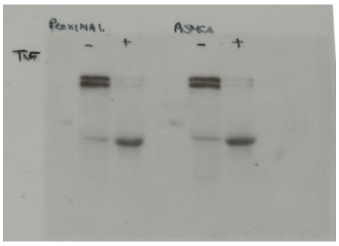


j) Supplement Fig 2c_alpha tubulin

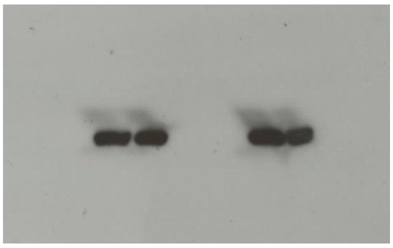

**Supplementary Figure 28. Uncropped western blots for Supplementary Figures 1a, 1b, 2a, 2b and 2c.** The relevant figures are indicated in the blot titles.

a) Supplement Fig 3e_BMPR-II

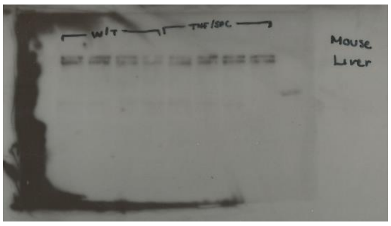


b) Supplement Fig 3e_alpha tubulin

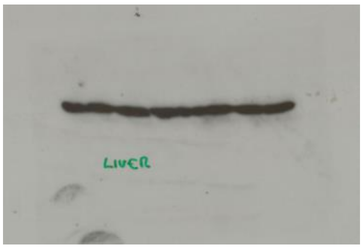


c) Supplement Fig 4a_MYC

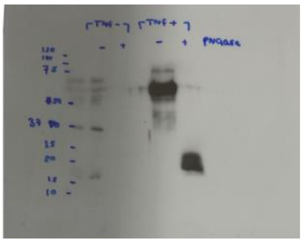

d) Supplement Fig 4a_Coomassie

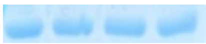

e) Supplement Fig 4c_BMPR-II IB:MYC

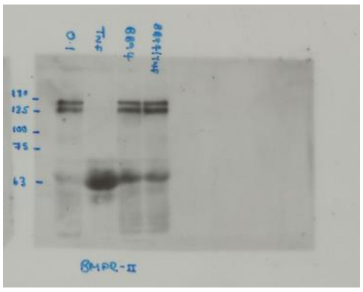

f) Supplement Fig 4c_alpha tubulin

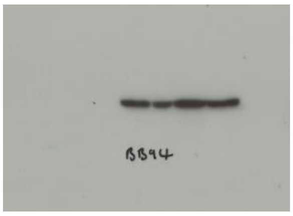


g) Supplement Fig 4d_WCL:MYC

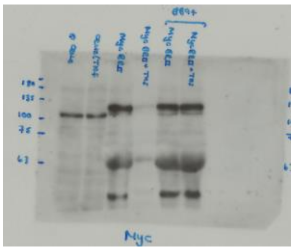


h) Supplement Fig 4d WCL:BMPR-II

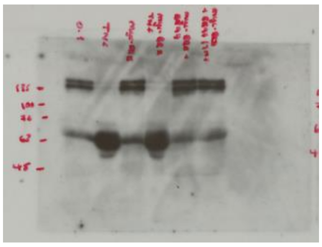

i) Supplement Fig 4d_alpha tubulin

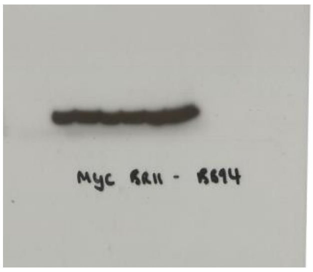

j) Supplement Fig 4d_IP:BMPR-II

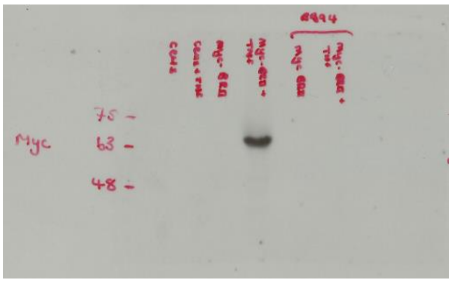


**Supplementary Figure 29. Uncropped western blots for Supplementary Figures 3e, 4a, 4c and 4d.** The relevant figures are indicated in the blot titles.

a) Supplement Fig 5c_ADAM10

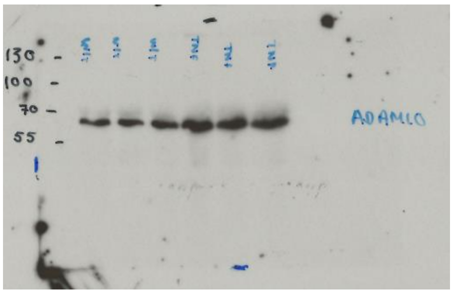


b) Supplement Fig 5c_ADAM17

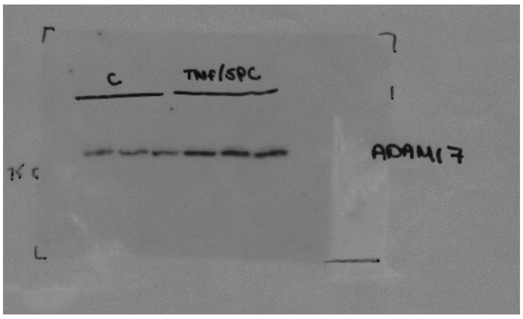


c) Supplement Fig 5c_beta actin

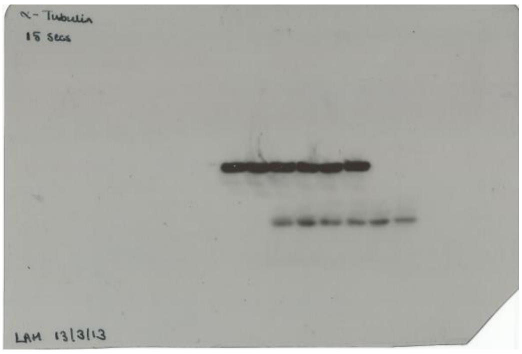


d) Supplement Fig 5e_BMPR-II

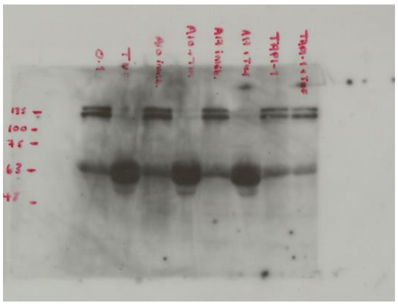

e) Supplement Fig 5e_alpha tubulin

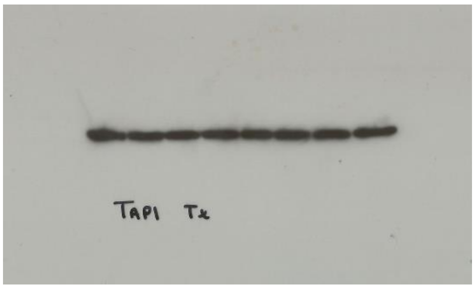


f) Supplement Fig 5e_IP:BMPR-II IB:MYC

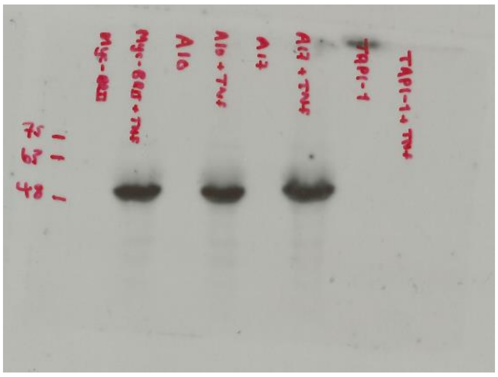


**Supplementary Figure 30. Uncropped western blots for Supplementary Figures 5c and 5e.** The relevant figures are indicated in the blot titles.

a) Supplement Fig 6b_WCL:MYC (left panel)

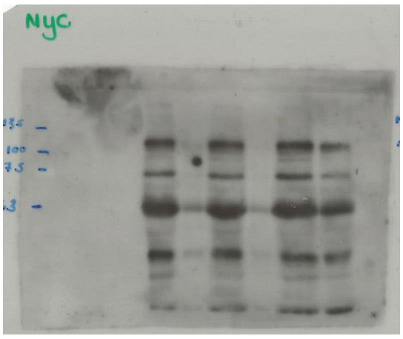


b) Supplement Fig 6b_BMPR-II (left panel)

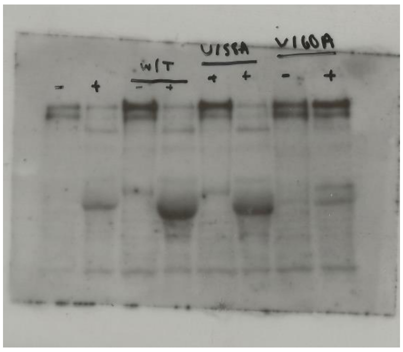

c) Supplement Fig 6b_alpha tubulin (left panel)

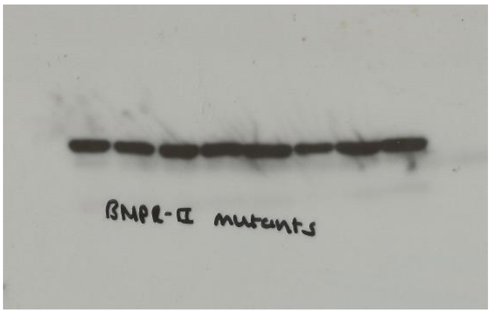


d) Supplement Fig 6b_IB:MYC (left panel)

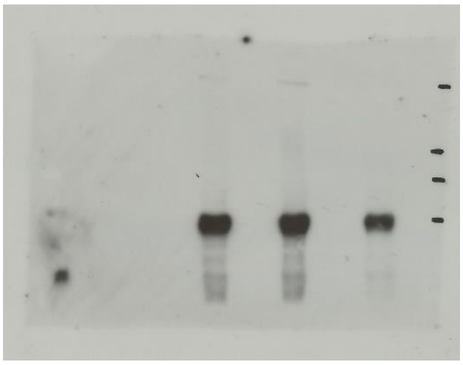

e) Supplement Fig 6b_WCL:MYC (right panel)

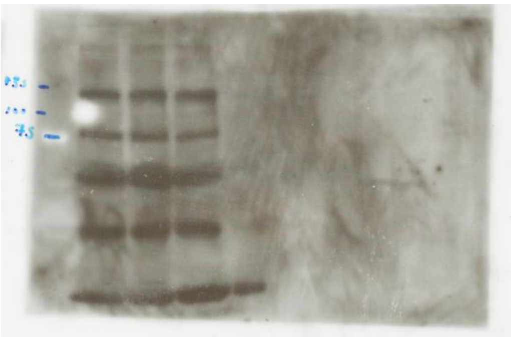

f) Supplement Fig 6b_BMPR-II (right panel)

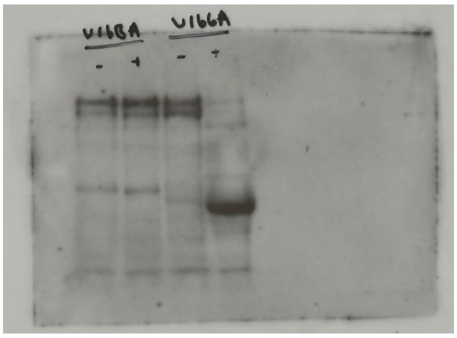

g) Supplement Fig 6b_alpha tubulin (right panel)

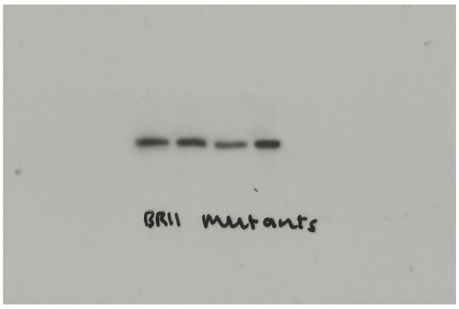


h) Supplement Fig 6b_IB:MYC (right panel)

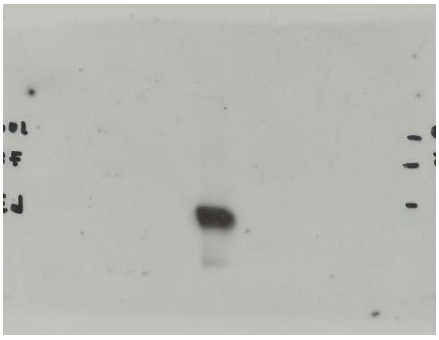

**Supplementary Figure 31. Uncropped western blots for Supplementary Figures 5c and 5e.** The relevant figures are indicated in the blot titles.

a) Supplement Fig 7a_BMPR-II (left panel)

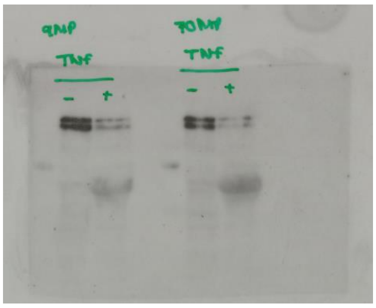


b) Supplement Fig 7a_alpha tubulin (left panel)

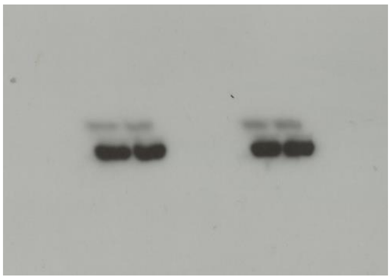

c) Supplement Fig 7a_BMPR-II (right panel)

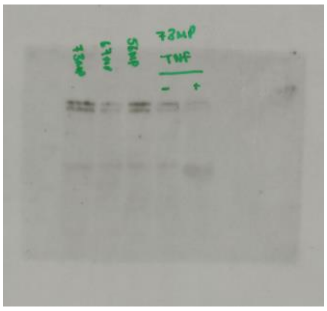


d) Supplement Fig 7a_alpha tubulin (right panel)

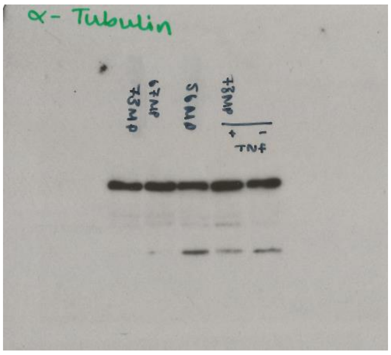


**Supplementary Figure 32. Uncropped western blots for Supplementary Figures 7a.** The relevant figures are indicated in the blot titles.

a) Supplement Fig 7b_pSMAD1/5 (left panel)

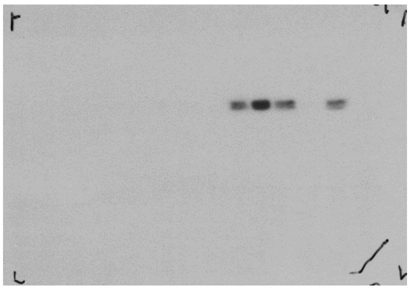

b) Supplement Fig 7b_tSMAD1 (left panel)

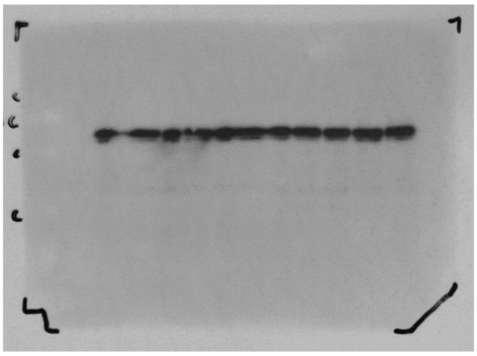

c) Supplement Fig 7b_alpha tubulin (left panel)

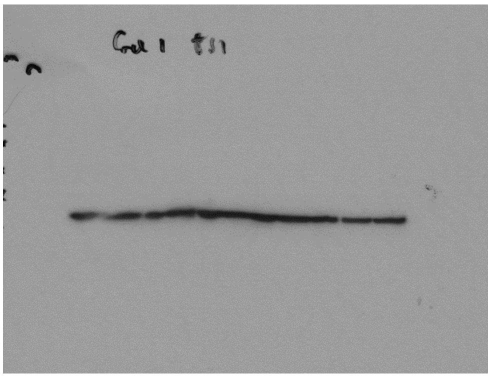

d) Supplement Fig 7b_pSMAD1/5 (right panel)

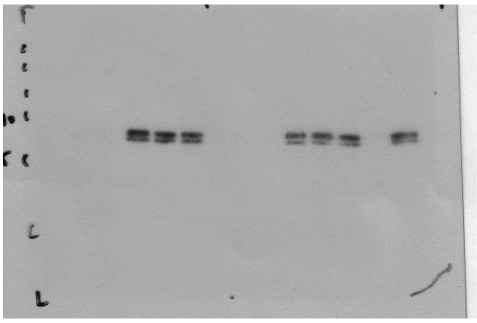

e) Supplement Fig 7b_tSMAD1 (right panel)

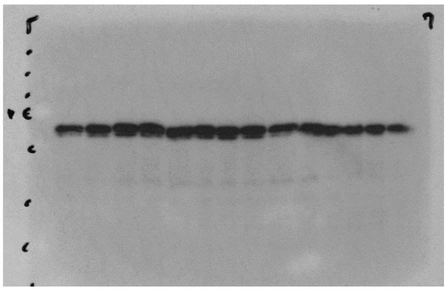

f) Supplement Fig 7b_alpha tubulin (right panel)

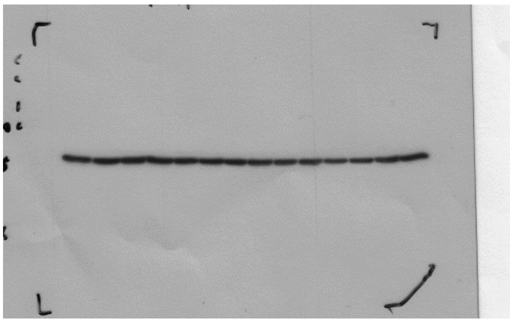

g) Supplement Fig 7c_BMPR-II

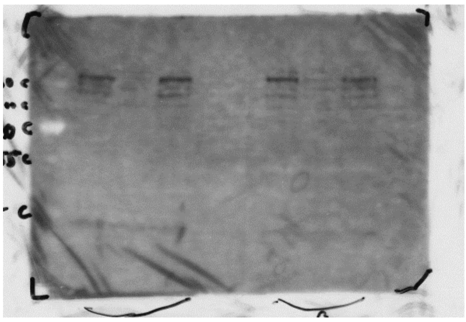

h) Supplement Fig 7c_alpha tubulin

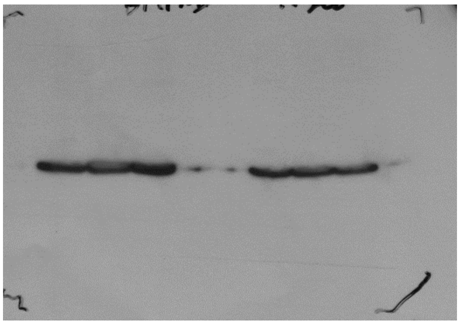

**Supplementary Figure 33. Uncropped western blots for Supplementary Figures 7b and 7c.** The relevant figures are indicated in the blot titles.

a) Supplement Fig 7d_pSMAD1/5 (right panel)

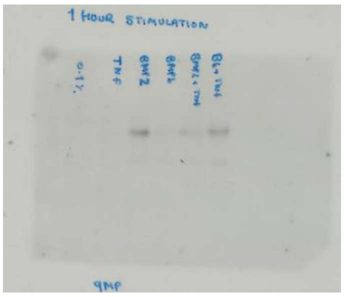


e) Supplement Fig 7d_pSMAD1/5 (left panel)

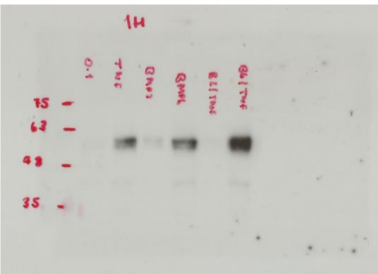


b) Supplement Fig 7d_tSMAD1 (right panel)

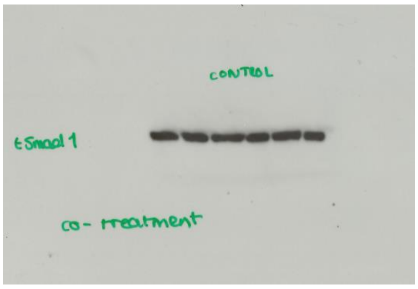


f) Supplement Fig 7d_tSMAD1 (left panel)

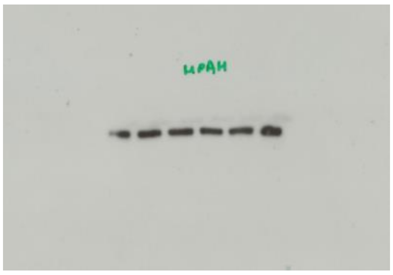


c) Supplement Fig 7d_ID1 (right panel)

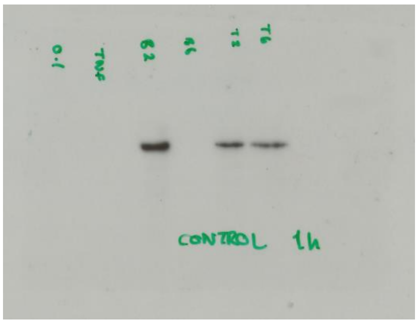


g) Supplement Fig 7d_ID1 (left panel)

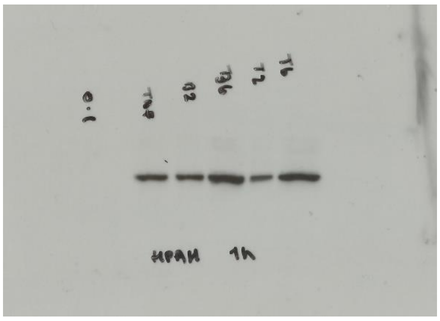


d) Supplement Fig 7d_alpha tubulin (right panel)

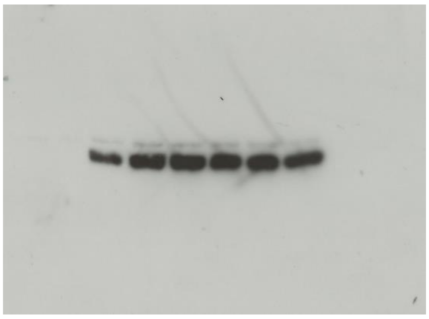

h) Supplement Fig 7d_alpha tubulin (left panel)

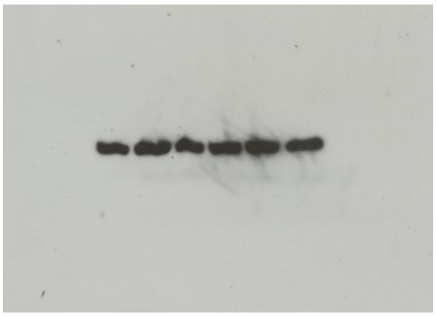

**Supplementary Figure 34. Uncropped western blots for Supplementary Figure 7d**. The relevant figures are indicated in the blot titles.

a) Supplement Fig 11a_NOTCH1

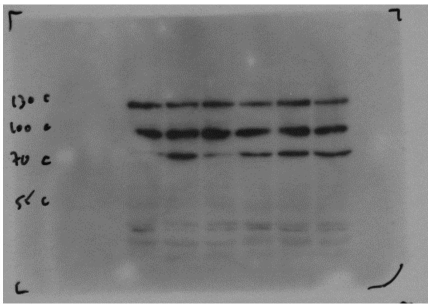

c) Supplement Fig 11a_NOTCH3

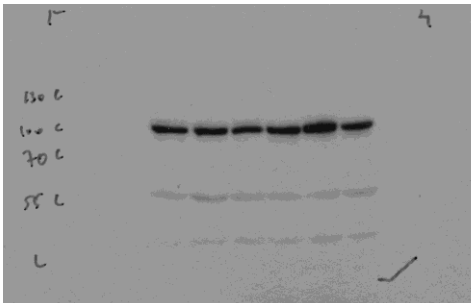

b) Supplement Fig 11a_NOTCH2

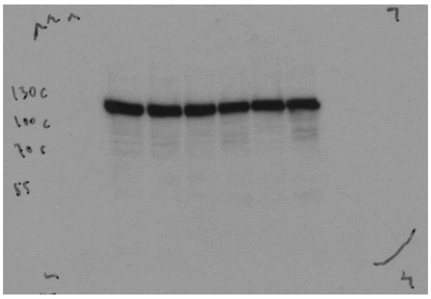

d) Supplement Fig 11a_alpha tubulin

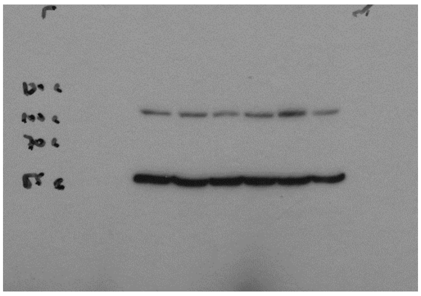

**Supplementary Figure 35. Uncropped western blots for Supplementary Figure 11a**. The relevant figures are indicated in the blot titles.

a) Supplement Fig 11c_NOTCH1 (left panel)

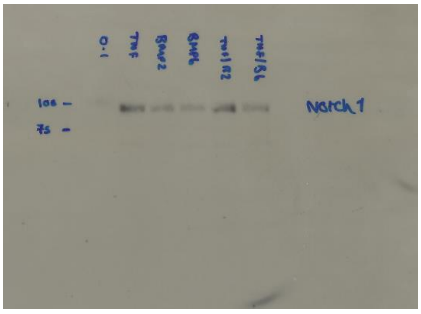


e) Supplement Fig 11c_NOTCH1 (right panel)

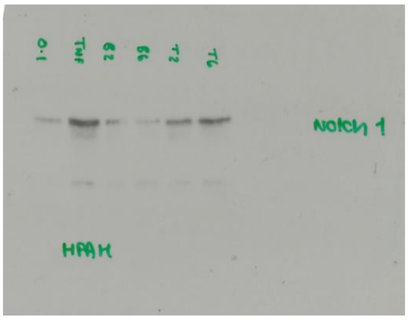


b) Supplement Fig 11c_NOTCH2 (left panel)

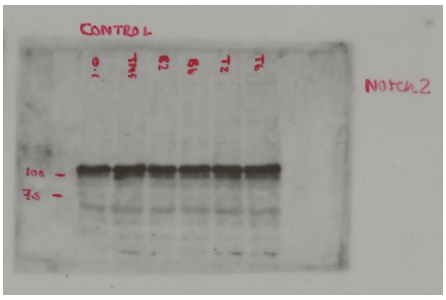


f) Supplement Fig 11c_NOTCH2 (right panel)

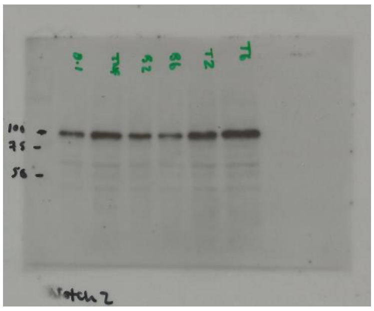


c) Supplement Fig 11c_NOTCH3 (left panel)

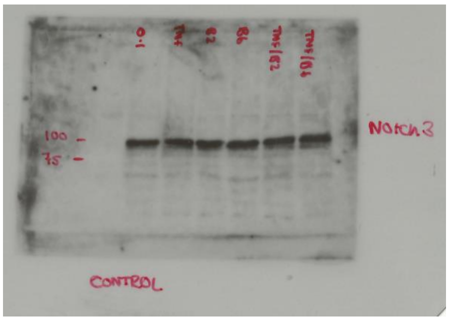


g) Supplement Fig 11c_NOTCH3 (right panel)

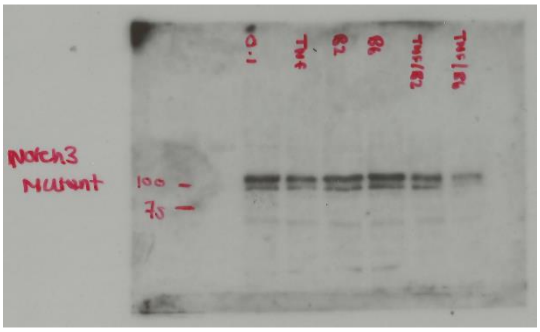


d) Supplement Fig 11c_alpha tubulin (left panel)

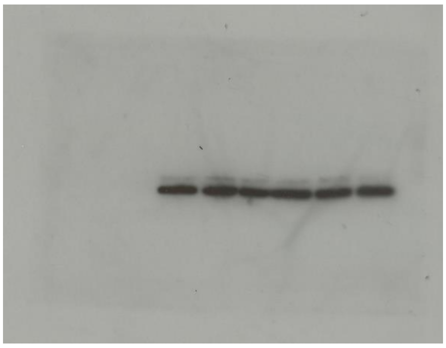

h) Supplement Fig 11c_alpha tubulin (right panel)

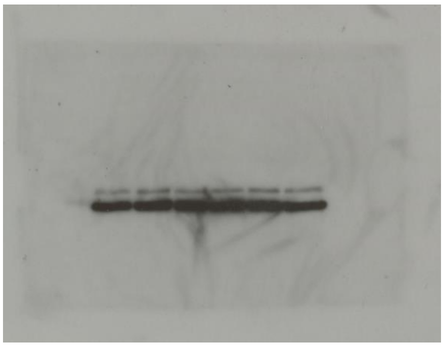

**Supplementary Figure 36. Uncropped western blots for Supplementary Figure 11c**. The relevant figures are indicated in the blot titles.

a) Supplement Fig 13g_NOTCH2 panel)

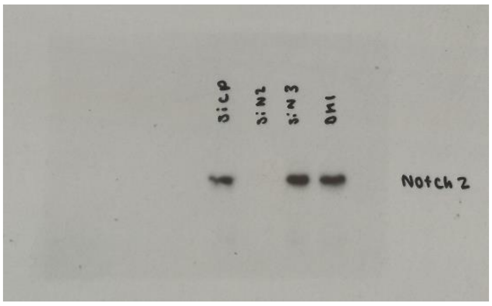


b) Supplement Fig 13g_NOTCH3

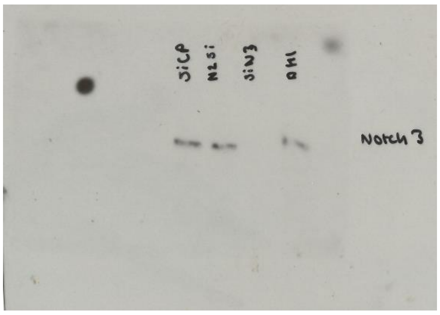


c) Supplement Fig 13g_alpha tubulin

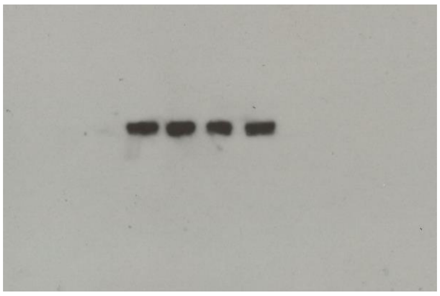

d) Supplement Fig 14d_NOTCH1 (left

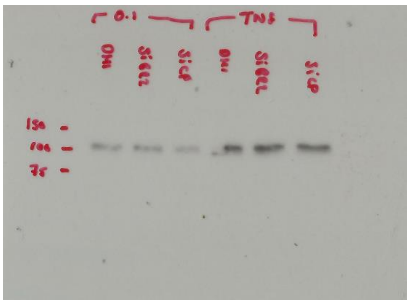

e) Supplement Fig 14d_alpha tubulin (left panel)

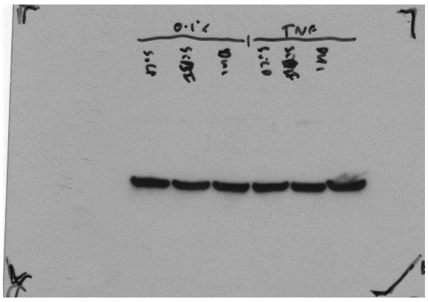

f) Supplement Fig 14d_NOTCH1 (right panel)

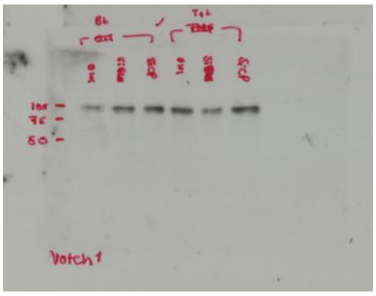


g) Supplement Fig 14d_alpha tubulin (right panel)

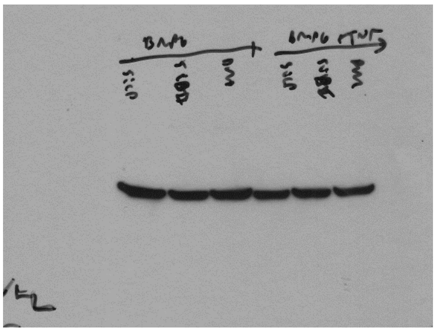

**Supplementary Figure 37. Uncropped western blots for Supplementary Figures 13g and 14d**. The relevant figures are indicated in the blot titles.

a) Supplement Fig 14e_NOTCH1 (left panel)

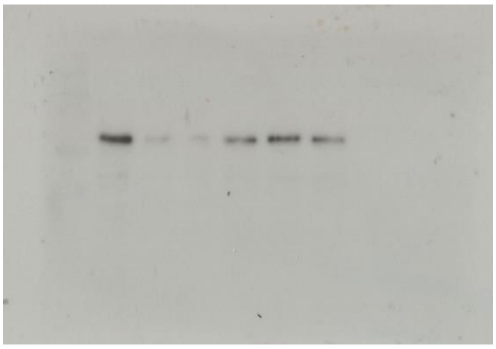

c) Supplement Fig 14e_NOTCH1 (right panel)

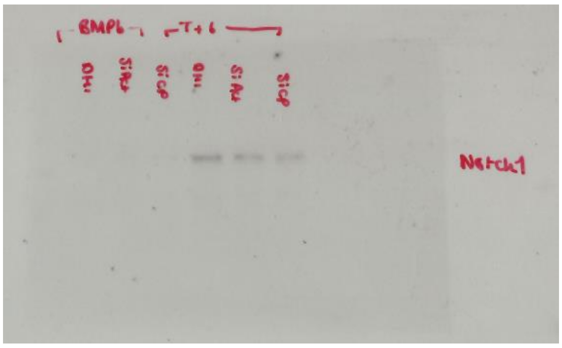


b) Supplement Fig 14e_alpha tubulin (left panel)

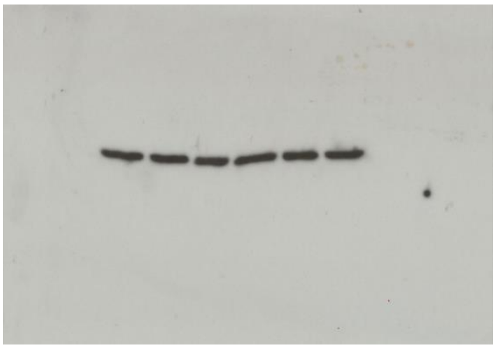

d) Supplement Fig 14e_alpha tubulin (right panel)

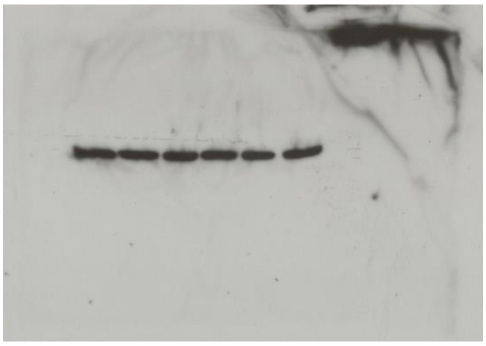

**Supplementary Figure 38. Uncropped western blots for Supplementary Figure 14e**. The relevant figures are indicated in the blot titles.

a) Supplement Fig 14f_NOTCH2 (left panel)

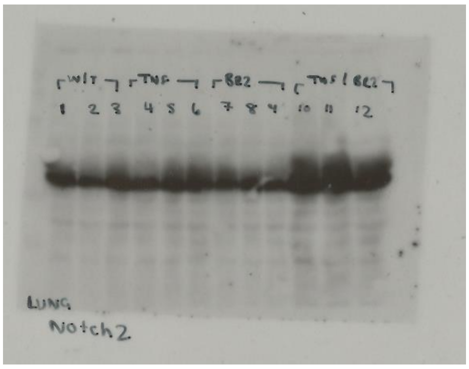


b) Supplement Fig 14f_NOTCH3 (left panel)

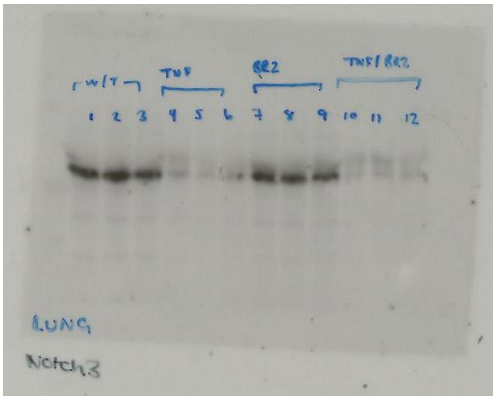


c) Supplement Fig 14f_alpha tubulin (left panel)

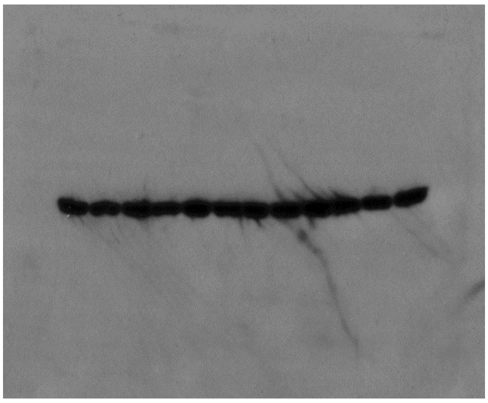

d) Supplement Fig 14f_NOTCH2 (right panel)

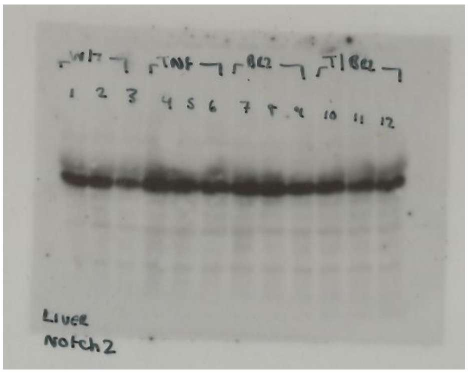


e) Supplement Fig 14f_NOTCH3 (right panel)

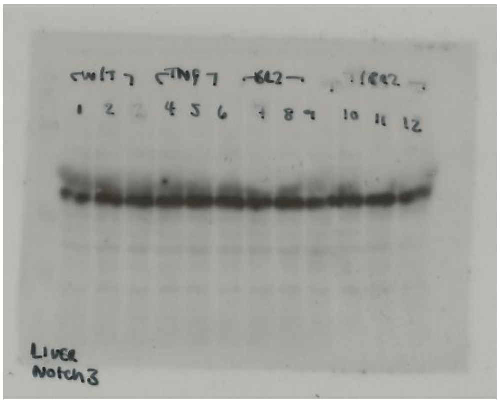


f) Supplement Fig 14f_alpha tubulin (right panel)

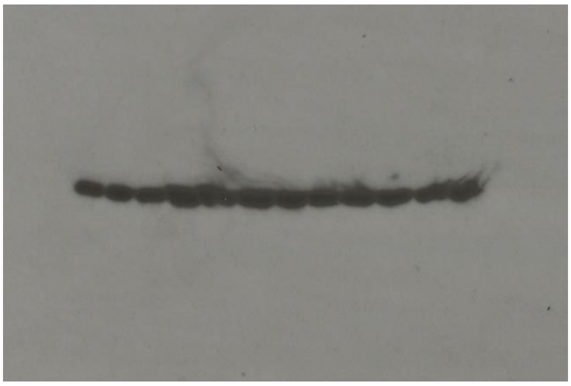

**Supplementary Figure 39. Uncropped western blots for Supplementary Figure 14f.** The relevant figures are indicated in the blot titles.

**Supplementary Table 1**: Left ventricular function in the Sugen-hypoxia rat model

| | | **Sugen + Hypoxia - 11 weeks** | |
|---|---|---|---|
| | **Control** | **Vehicle** | **Etanercept** |
| **n** | 6 | 7 | 8 |
| **Heart rate (bpm)** | 445.3 ± 22.4 | 425.5 ± 45.9 | 373.1 ± 68.4* |
| **LVESP (mmHg)** | 107.4 ± 10.7 | 104.5 ± 22.8 | 105.3 ± 14.5 |
| **LVEDP (mmHg)** | 21.59 ± 9.78 | 14.45 ± 8.36 | 17.18 ± 7.85 |
| **Stroke Volume (RVU)** | 19.23 ± 4.30 | 22.38 ± 5.34 | 23.04 ± 8.75 |
| **Cardiac Output (RVU/min)** | 8581 ± 2030 | 9450 ± 2360 | 8253 ± 2471 |
| **dP/dt max (mmHg $s^{-1}$)** | 6835 ± 799 | 6363 ± 1574 | 6470 ± 1084 |
| **Blood Pressure (mmHg)** | 100.0 ± 3.69 | 101.0 ± 6.90 | 100.6 ± 10.0 |

+/- SEM, *$P$<0.05 relative to control
LVESP: Left Ventricular End-Systolic Pressure
LVEDP: Left Ventricular End-Diastolic Pressure
RVU: Relative volume units

**Supplementary Table 2:** Pulmonary arterial smooth muscle cell clinical information

| **Subject ID** | **Sex** | **Age** | ***BMPR2* Mutation** | **Clinical Details** |
|---|---|---|---|---|
| **Control PASMCs** | | | | |
| 9MP | M | 72 | - | Adenocarcinoma |
| 32MP | F | 58 | - | Emphysema |
| 70MP | M | 66 | - | Squamous cell carcinoma |
| **HPAH PASMCs** | | | | |
| 56MP | M | 58 | C347R | FPAH; transplant |
| 67MP | M | 22 | W9X | FPAH; transplant |
| 73MP | F | 30 | R899X | FPAH; transplant |

**Supplementary Table 3. Mutagenesis primers for myc-tagged BMPR-II valine-alanine plasmids.**

| **Mutation** | **Forward** | **Reverse** |
|---|---|---|
| V158A | AATAATCATTGCTTTGGCATCAG**C**CTCTGTATTAGCTGTTTTGATAG | CTATCAAAACAGCTAATACAGAG**G**CTGATGCCAAAGCAATGATTATT |
| V160A | TGCTTTGGCATCAGTCTCTG**C**ATTAGCTGTTTTGATAGTTG | CAACTATCAAAACAGCTAAT**G**CAGAGACTGATGCCAAAGCA |
| V163A | CATCAGTCTCTGTATTAGCTG**C**TTTGATAGTTGCCTTATGCTT | AAGCATAAGGCAACTATCAAA**G**CAGCTAATACAGAGACTGATG |
| V166A | GTCTCTGTATTAGCTGTTTTGATAG**C**TGCCTTATGCTTTGGA | TCCAAAGCATAAGGCA**G**CTATCAAAACAGCTAATACAGAGAC |

## Supplementary Table 4. Quantitative RT-PCR primers for human.

| Target | Forward | Reverse |
|---|---|---|
| *ACTB* | GCACCACACCTTCTACAATGA | GTCATCTTCTCGCGGTTGGC |
| *ALK3* | TTCGTATGACGGATCACTCG | AGCCCTACATCATGGCTGAC |
| *ADAM10* | TTCTGCTCCTCTCCTGGGCGG | GCTCTTTTGGCACGCTGGTGT |
| *ADAM12* | CACGGCAGAGGGGTGTGCAA | TCCGGCAGCAAGAAGACACAGG |
| *ADAM15* | GGCACTGAGGAGCAGCAGGC | CGGCCTGGGACCAACTCCCT |
| *ADAM17* | TAGCAGTGAGTGCCCGCCTCC | CATAGGGCACACAGCGGCCAG |
| *B2M* | CTCGCGCTACTCTCTCTTTCT | CATTCTCTGCTGGATGACGTG |
| *BMPR2* | CAAATCTGTGAGCCCAACAGTCAA | GAGGAAGAATAATCTGGATAAGGACCAAT |
| *FYN* | GGCGGGGAGAGGACCATGTGAG | GAGGCAGGACTGGTCTTTTTCCACG |
| *HES1* | GGAATCCCCCGTCTACCTCT | TGAGCAAGTGCTGAGGGTTT |
| *HEY1* | GGCTCTAGGTTCCATGTCCC | AGCAGATCCCTGCTTCTCAA |
| *HEY2* | GCCATACAGATGCCGACAGA | CAGTTACCGAGCTGCCTTGA |
| *ID1* | GACGGCCGAGGCGGCATG | GGGGAGACCCACAGAGCACG |
| *NOTCH3* | CCTAGACCTGGTGGACAA | ACACAGTCGTAGCGGTTG |
| *MMP14* | CGGACCATGTCTCCCGCCCC | GTGACTGGGGTGAGCGCTGTG |
| *MMP15* | CTTGGCGTAGCGGCCGAAGA | CTGGGCGGAACGCATGGTGG |
| *MMP16* | ACCTTCCACCGACTGACCCCA | GGTACACCGCATCGGGGCTTC |
| *MMP17* | GACAAGGTGCGCGTCTGGCA | CTGGGCCACCGCGTCAAAGT |
| *MMP24* | TCGCTGGTTCTGGCGTCTGC | GGGTACCCAGGCTCCACCGT |
| *SRC* | TCCACCGGGACCTTCGTGCA | AATTTGGCACCTTGCCGCGC |
| *YES* | GCGGCCGGAGGACAGATTTGAT | ACGGACATGGTGACACTGTAGTGGG |

## Supplementary Table 5. Quantitative RT-PCR primers for mouse

| Target | Forward | Reverse |
|---|---|---|
| *Bmpr2* | AGATCTATCCTCTCCCTAAG | TTAGAATGGACTGCCCTGTC |
| *Notch1* | GTCGCAACTGTGAGAGTGA | TCGCAGAAGGCTGTGTTGAT |
| *Notch2* | AGCAGACTGGATGAACCGTG | AGCTGGAAAGTCACGATGGG |
| *Notch3* | GACTGCTCACTGAACGTGGA | CACACCGGCTGTTGTTGAAG |

## Supplementary Table 6. Quantitative RT-PCR primers for rat

| Target | Forward | Reverse |
|---|---|---|
| *Actb* | CTGCCTGACGGTCAGGT | TGGATGCCACAGGATTCCAT |
| *Alk2* | TCTGTGCTAATGATGATGGCTCTCC | TTCGCGATCCAGGGAAGGATTTC |
| *Acvr2a* | ACTGCTGCAGATGGACCTG | AGCTCCAGTTCAGAGTCCC |
| *Bmp6* | AGCACAGAGACTCTGACCTATTTTTG | CCACAGATTGCTAGTTGCTGTGA |
| *Hes1* | CGACACCGGACAAACCAAA | GAATGTCTGCCTTCTCCAGCTT |
| *Hey1* | AAGCTGAGATCTTGCAGATG | GGCGGCGACAGTTTGGAG |
| *Hey2* | TGACATCCTCCATGTCCC | ACTGATAACGGTGGGCTG |
